# Epigenetic control of skeletal muscle atrophy

**DOI:** 10.1186/s11658-024-00618-1

**Published:** 2024-07-08

**Authors:** Wenpeng Liang, Feng Xu, Li Li, Chunlei Peng, Hualin Sun, Jiaying Qiu, Junjie Sun

**Affiliations:** 1 Key Laboratory of Neuroregeneration of Jiangsu and Ministry of Education, Co-Innovation Center of Neuroregeneration, NMPA Key Laboratory for Research and Evaluation of Tissue Engineering Technology Products, Nantong University, Nantong, 26001 China; 2 Department of Prenatal Screening and Diagnosis Center, Affiliated Maternity and Child Health Care Hospital of Nantong University, Nantong, 226001 China; 3 Department of Endocrinology, Affiliated Hospital 2 of Nantong University and First People’s Hospital of Nantong City, Nantong, 226001 China; 4 Nantong Center for Disease Control and Prevention, Medical School of Nantong University, Nantong, 226001 China; 5 Department of Medical Oncology, Tumor Hospital Affiliated to Nantong University, Nantong, 226000 China

**Keywords:** Skeletal muscle atrophy, epigenetic, Ubiquitin–proteasome, m6A, Histone modifications

## Abstract

Skeletal muscular atrophy is a complex disease involving a large number of gene expression regulatory networks and various biological processes. Despite extensive research on this topic, its underlying mechanisms remain elusive, and effective therapeutic approaches are yet to be established. Recent studies have shown that epigenetics play an important role in regulating skeletal muscle atrophy, influencing the expression of numerous genes associated with this condition through the addition or removal of certain chemical modifications at the molecular level. This review article comprehensively summarizes the different types of modifications to DNA, histones, RNA, and their known regulators. We also discuss how epigenetic modifications change during the process of skeletal muscle atrophy, the molecular mechanisms by which epigenetic regulatory proteins control skeletal muscle atrophy, and assess their translational potential. The role of epigenetics on muscle stem cells is also highlighted. In addition, we propose that alternative splicing interacts with epigenetic mechanisms to regulate skeletal muscle mass, offering a novel perspective that enhances our understanding of epigenetic inheritance’s role and the regulatory network governing skeletal muscle atrophy. Collectively, advancements in the understanding of epigenetic mechanisms provide invaluable insights into the study of skeletal muscle atrophy. Moreover, this knowledge paves the way for identifying new avenues for the development of more effective therapeutic strategies and pharmaceutical interventions.

## Introduction

Skeletal muscle, constituting approximately 40–50% of the total body mass in a healthy individual, is the most abundant tissue in the human body. It is indispensable for movement, heat generation, and metabolic equilibrium. Beyond these functions, skeletal muscle acts as a critical regulator of the body’s protein reserves, glucose, and lipid homeostasis [1, 2]. Skeletal muscle atrophy, a condition characterized by a decrease in muscle mass and strength, can result from a variety of factors, including genetic mutations [e.g., Duchenne muscular dystrophy (DMD), amyotrophic lateral sclerosis (ALS), and spinal muscular atrophy (SMA)], denervation, disuse, cancer cachexia, weightlessness, aging, and chronic diseases such as diabetes [3–8]. This condition not only impairs an individual’s mobility but also significantly affects their quality of life, imposing a considerable economic and social burden. Histologically, skeletal muscle atrophy is marked by a reduction in muscle fiber cross-sectional area, disintegration of myofilament structures, degradation of myogenic fibers, mitochondrial dysfunction, and increased mitochondrial autophagy. Despite the vital role of skeletal muscle and the severe consequences of its atrophy, it remains one of the least effectively treated tissues [3, 4]. Furthermore, skeletal muscle exhibits secretory functions; following exercise or injury, it releases cytokines into the bloodstream, influencing systemic health [9]. Given these facts, the need for comprehensive research into the pathogenesis and therapeutic approaches for skeletal muscle atrophy is both urgent and significant.

Under normal physiological conditions, skeletal muscle anabolic and catabolic metabolism are in dynamic balance and work together to maintain muscle quality. Skeletal muscle atrophy occurs when this balance is disrupted, specifically when catabolic processes predominate, leading to greater protein breakdown than synthesis. The principal pathways involved in this degradation are the ubiquitin–proteasome pathway, the calpain system, and the autophagy-lysosome pathway. A significant indicator of muscle atrophy is the overactivation of the ubiquitin–proteasome degradation system, highlighted by the upregulation of E3 ubiquitin ligases such as muscle atrophy F-box (MAFbx, also known as Atrogin-1), Muscle RING Finger-1(MuRF1, also known as Trim63), and TNF receptor associated factor 6 (Traf6) [10–12], which can be triggered by factors such as proinflammatory cytokines, oxidative stress, and fluctuations in energy levels that then stimulate the ubiquitin–proteasome proteolytic system through various downstream signaling cascades. This process begins with the ubiquitin-activating enzyme E1, which attaches ubiquitin to ATP, forming an activated ubiquitin molecule. The E2 ubiquitin-conjugating enzyme then transfers this activated ubiquitin to the E3 ubiquitin ligase, which in turn links the ubiquitin to the target protein, marking it for degradation. The complex nature and not fully understood mechanisms underlying skeletal muscle atrophy have hindered the development of effective pharmacological interventions.

**Fig. 1:**
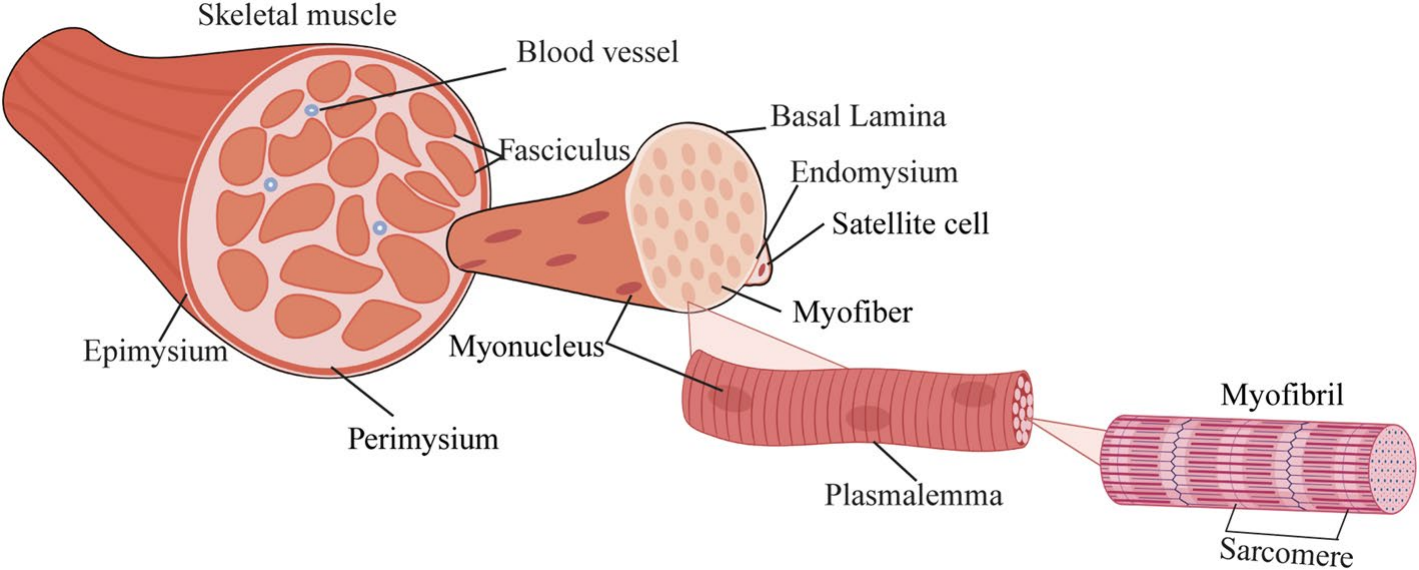
Organization of skeletal muscle. The epimysium surrounds the entire skeletal muscle. The next inner layer is the perimysium, which surrounds the fascicles and blood vessels. Below the perimysium, between the basal lamina and the endomysium, satellite cells are distributed. The endomysium surrounds individual myofibers, each of which contains myofibrils. These myofibrils are made up of sarcomeres. This figure was created with https://www.BioRender.com

Skeletal muscle is wrapped externally by the epimysium, and one layer inward is the perimysium, which surrounds the fascicles and blood vessels. Below the perimysium, satellite cells (SCs) are distributed between the basal lamina and the endomysium. The endomysium wraps around the muscle fibers and contains myofibers within each muscle fiber, which are made up of sarcomeres (Fig. 1). Individual muscle fibers constitute the smallest functional units of skeletal muscle. Based on the expression of myosin heavy chain (MyHC) isozymes, human skeletal muscle fibers are categorized into three types: type I, type IIa, and type IIx. Additionally, rodent skeletal muscles include type IIb fibers. Type I fibers, known as slow-twitch fibers, contract slowly and predominantly utilize oxidative phosphorylation for energy. In contrast, type IIb and IIx fibers, referred to as fast-twitch fibers, contract rapidly and primarily generate energy through the glycolytic pathway [13]. There are fibers that express multiple MyHC isoforms, called hybrid fibers. Hybrid fibers are common in humans and have variable proportions of MyHC isoforms that correspond to the high degree of muscle plasticity. Although both fast- and slow-twitch muscle fibers can undergo atrophy, the sensitivity to atrophy varies between these fiber types. Slow-twitch fibers are more prone to wasting and denervation-induced atrophy, while fast-twitch fibers are particularly vulnerable to atrophy associated with conditions such as cancer cachexia, diabetes, and aging [14–16]. The differential susceptibility of muscle fiber types to various forms of atrophy has been a subject of long-standing investigation. Despite extensive research, the specific atrophy signaling pathways that mediate these differences between fast and slow muscles remain debatable.

As a resident stem cell, the fate of SCs is critical for skeletal muscle injury repair. Under homeostatic conditions, SCs remain quiescent for extended periods of time. Upon receiving activation signals, SCs promptly exit their dormant state and commence cell cycle progression to proliferate. The proliferating SCs subsequently undergo differentiation to repair damaged tissue, while a subset of SCs reenters quiescence following self-renewal, maintaining a stable stem cell population [17]. This dynamic transition between states underpins the plasticity of skeletal muscle and constitutes a crucial mechanism for triggering regeneration following muscle atrophy. The transcription factor *Paired-box 7* (*Pax7*) plays a pivotal role in sustaining SC quiescence and is instrumental in regulating both self-renewal and proliferation [18]. The myogenic regulatory factors (MRFs) family of transcription factors, including *Myogenic Differentiation 1* (*MyoD* or *MyoD1*), *Myogenin* (*Myog*), *Myogenic Factor 5*** (***Myf5*), and *Myogenic Regulatory Factor 4* (*MRF4*, also known as *Myf6*), act as molecular switches that determine the fate of SCs and skeletal muscle phenotype establishment [19, 20]. Discovering the drivers of MRF expression and elucidating the molecular mechanisms could help to understand the skeletal muscle atrophy process.

Increasing evidence indicates that epigenetic inheritance plays an important role in determining muscle stem cell fate and influencing the progression of skeletal muscle atrophy. Epigenetics involves the regulation of gene expression via chemical modifications to histones, DNA, or RNA, without altering the underlying gene sequence, thereby impacting the phenotype [21, 22]. The establishment and removal of these chemical marks are regulated by various epigenetic regulatory proteins, categorized as readers, writers, and erasers. Although genetic studies on various epigenetic regulatory proteins have affirmed their significance in maintaining skeletal muscle quality, comprehensive investigations into the underlying mechanisms remain sparse [23, 24]. Recent advancements in high-throughput sequencing technologies have propelled the field of epigenetics into detailed molecular investigations through approaches such as epigenomics, epitranscriptomics, proteomics, and single-cell epitranscriptomics. A recent study has demonstrated alterations in chromatin accessibility during skeletal muscle atrophy at the resolution of individual muscle fibers, offering novel insights into the epigenetic regulation of skeletal muscle atrophy [25].

Here, we summarize and provide a mechanistic perspective gained from recent research on the epigenetic control of skeletal muscle atrophy. First, we outline in turn the different types of modifications of DNA, histones, and RNA underpinning the molecular basis of skeletal muscle atrophy. Their dynamics during skeletal muscle atrophy, their role in skeletal muscle atrophy, and their role in satellite cells are of major interest. We discuss the molecular mechanisms of epigenetic regulation of skeletal muscle atrophy at the transcriptional, post-transcriptional, and post-translational levels and summarize the key downstream molecules. In addition, the co-regulation of skeletal muscle atrophy by alternative splicing and epigenetic crosslinking is an interesting new mechanism. Finally, we discuss neglected but important directions in current study and the therapeutic potential of epigenetic drugs for skeletal muscle atrophy.

## The role of epigenetic in skeletal muscle atrophy

Epigenetic and epitranscriptomic modifications play crucial roles in the regulation of gene expression across transcriptional, post-transcriptional, and translational stages. These modifications, which include the addition or removal of molecular marks such as DNA methylation, histone modifications (e.g., acetylation, methylation), and RNA modifications (e.g., methylation, splicing), can either activate or inhibit gene expression, influencing cellular states and behaviors. In the context of disease, these epigenetic changes can persist through cell division, leading to long-lasting impacts on cellular functions and characteristics. Understanding epigenetic mechanisms provides valuable insights into the regulation of genes associated with muscle atrophy and the adaptative responses of muscle cells to functional demands. This knowledge opens new perspectives for exploring the origins and progression of skeletal muscle atrophy, offering novel targets for therapeutic intervention and management.

### DNA modifications

#### DNA modifications and regulators

Chemical modifications of DNA bases were discovered as early as 1948, with DNA methylation being the first to be recognized [26]. Various forms of DNA methylation exist, such as 5-methylcytosine (5mC), N^6^-methyladenine (6 mA), N^4^-methylcytosine (4mC), and 5-hydroxymethylcytosine (5hmC) [27]. In prokaryotes and lower eukaryotes, 6mA methylation prevails, whereas 4mC methylation is found in certain bacteria. 5hmC is generally considered a reactive intermediate in the process of DNA demethylation and has been shown to play a pivotal role in this process. In mammals, 5mC constitutes the predominant form of DNA methylation.

DNA methylation is catalyzed by a family of DNA methyltransferases (DNMTs). In this process, *S*-adenosylmethionine (SAM) acts as a methyl donor, transferring the methyl group to the fifth carbon atom of cytosine through a covalent bond [28, 29]. The DNMT family in mammals comprises three main members: DNMT1, DNMT2, and DNMT3 (Table 1). DNMT1 acts as a maintenance methyltransferase, preserving DNA methylation patterns during DNA replication. DNMT2, despite possessing methyltransferase capabilities, is unique in that it targets tRNA rather than DNA owing to the absence of an N-terminal regulatory region, thus not contributing to DNA methylation [30]. DNMT3A and DNMT3B are key players in the de novo methylation of DNA, targeting unmethylated DNA on both strands [31, 32] (Table 1). These enzymes also play roles in maintaining methylation patterns and methylating repetitive sequences [33]. DNMT3C, a rodent-specific duplicate of DNMT3B, shares structural similarities with DNMT3A and DNMT3B but lacks the PWWP domain in its N-terminal regulatory region, which is essential for chromatin binding. The primary role of DNMT3C is to methylate and, thus, inhibit the activation of evolutionarily recent retrotransposons during sperm development, safeguarding genomic integrity [34].

**Table 1:** List of DNA modification regulators

DNA modifications	Enzymes	
DNA methylation	DNMT	DNMT1
		DNMT3A and DNMT3B
DNA demethylation	TET	TET1
		TET2
		TET3

DNA methylation and demethylation processes work together to maintain a dynamic balance in cells, ensuring genome stability. The demethylation of 5-methylcytosine (5mC) involves the action of the ten-eleven translocation (TET) family of enzymes, which includes TET1, TET2, and TET3 (Table 1). These enzymes catalyze the oxidation of 5mC into three intermediate forms: 5hmC, 5-formylcytosine (5fC), and 5-carboxylcytosine (5caC). Following this, the intermediate products undergo a thymine DNA glycosylase (TDG)-mediated base excision repair process, leading to the conversion of 5mC back to unmodified cytosine (C)[35].

#### DNA methylation dynamic in skeletal muscle atrophy

Skeletal muscle tissue or cells originating from skeletal muscle display distinct methylation profiles compared with other tissues [36, 37]. These muscle-specific methylation patterns highlight a notable feature: genes coding for proteins predominantly found in skeletal muscle—such as *OBSCN*, *MYOT*, and *MYH7*, which play important roles in sarcomere organization—are found to be hypomethylated in skeletal muscle [38–40]. This hypomethylation of genes encoding dominant muscle proteins suggests a reduced susceptibility to regulatory mechanisms, thereby promoting their consistent expression essential for the fundamental functions of skeletal muscle, including contraction. Nonetheless, the relationship between these methylation patterns and muscle atrophy remains to be elucidated.

As we age, skeletal muscles atrophy and lose strength and function, leading to sarcopenia. A genome-wide analysis on methylation patterns showed an increase in methylation within the skeletal muscles of older people and in rodent models [41–43]. One gene significantly affected by this methylation process is *tubulin folding cofactor D* (*TBCD*), which plays a crucial role in microtubule assembly and is observed predominantly in samples from older humans [41]. Interestingly, most of these methylation sites are found within the gene’s body, not in the promoter regions, correlating less with gene expression levels. Similar methylation alterations have been identified in the skeletal muscles of aged rats, aligning with human findings. Notably, there is a reported increase in *Dnmt3b* expression in these cases [44]. A comprehensive meta-analysis conducted by Sarah Voisin et al. examined age-related DNA methylation changes in human skeletal muscle, pooling data from ten separate studies. This analysis uncovered 6710 distinct methylation regions across 6367 unique genes. The findings indicated a significant increase in DNA methylation at polysome target genes and bivalent chromatin domains, whereas methylation at enhancer regions was significantly higher [45]. To facilitate broader access and analysis of these findings, the research team has introduced MetaMeth, a web-based tool designed for user-friendly interaction and efficient data sharing (https://sarah-voisin.shinyapps.io/MetaMeth/).

Progressive weight-bearing exercise has been shown to partially reverse the hypermethylation observed in aged skeletal muscle [46]. Notably, the changes in methylation and demethylation patterns following exercise are more significant in elderly individuals than in younger counterparts, suggesting that the benefits of exercise for skeletal muscle health and function may be more pronounced in older adults [47].

Compared with aging skeletal muscles, there has been limited systematic analysis on DNA methylation in other models of muscle atrophy. The question of whether skeletal muscle atrophy induced by different factors shares DNA methylation patterns remains open. Fisher et al. developed a denervated muscle atrophy model in rats by injecting tetrodotoxin (TTX) into the peripheral nerves [48] and used real-time quantitative PCR and pyrosequencing to investigate DNA methylation in the promoter regions of several atrophy-related genes (atrogenes) and discovered that *Myog*, *MuRF1*, *MAFbx*, and *Chrna1* exhibited demethylation at critical points of denervated muscle atrophy, corresponding to increased gene expression. Upon cessation of TTX treatment and recovery of the skeletal muscles, the methylation status of these genes was significantly reversed, coinciding with reduced gene expression. The findings of Fisher et al. indicate that DNA methylation changes play a crucial role in atrogene activation. Additionally, muscle atrophy resulting from prolonged bed rest was examined by Lisa Van Dyck et al. in a study involving critically ill adult patients in the intensive care unit [49]. They identified two regions of hypomethylation associated with muscle regeneration and postsynaptic acetylcholine receptors in the patients.

#### The roles of DNA methylation regulators in skeletal muscle atrophy

In mouse models of denervation, aging, and disuse-induced muscle atrophy, a significant downregulation of *Dnmt3a* has been observed, while the levels of the other two methyltransferases, *Dnmt1* and *Dnmt3b*, remain relatively unchanged [50]. This finding contrasts with the observed hypermethylation in aged skeletal muscle, implying the involvement of alternative regulators of DNA methylation or different mechanisms behind the increase in methylation [51]. Notably, *Dnmt3a* knockout (KO) mice demonstrated a substantial reduction in muscle regeneration capability following injury, with SCs appearing smaller (in terms of length and area) and exhibiting inhibited myotube differentiation [50]. SCs from mice with a conditional KO of *Dnmt3a*, driven by the *Pax3* promoter (specific to muscle SCs), also displayed a marked decline in proliferation when cultured [52]. At the molecular level, *Dnmt3a* was shown to enhance the expression of *growth differentiation factor 5* (*Gdf5*) and *p57Kip2*, a member of the Cip/Kip family of cyclin-dependent kinase inhibitors, through DNA methylation-dependent pathways [50, 52]. Intriguingly, compared with mice with a complete KO of *Dnmt3a* (induced by Cre recombinase under the human α-actin promoter), those with a muscle satellite cell-specific deletion of *Dnmt3a* exhibited more severe muscle mass loss. These findings collectively suggest that *Dnmt3a*-mediated DNA methylation changes play a crucial role not only in the proliferation and differentiation of muscle SCs but also in influencing various aspects of skeletal muscle atrophy, potentially impacting other cell types as well.

**Fig. 2:**
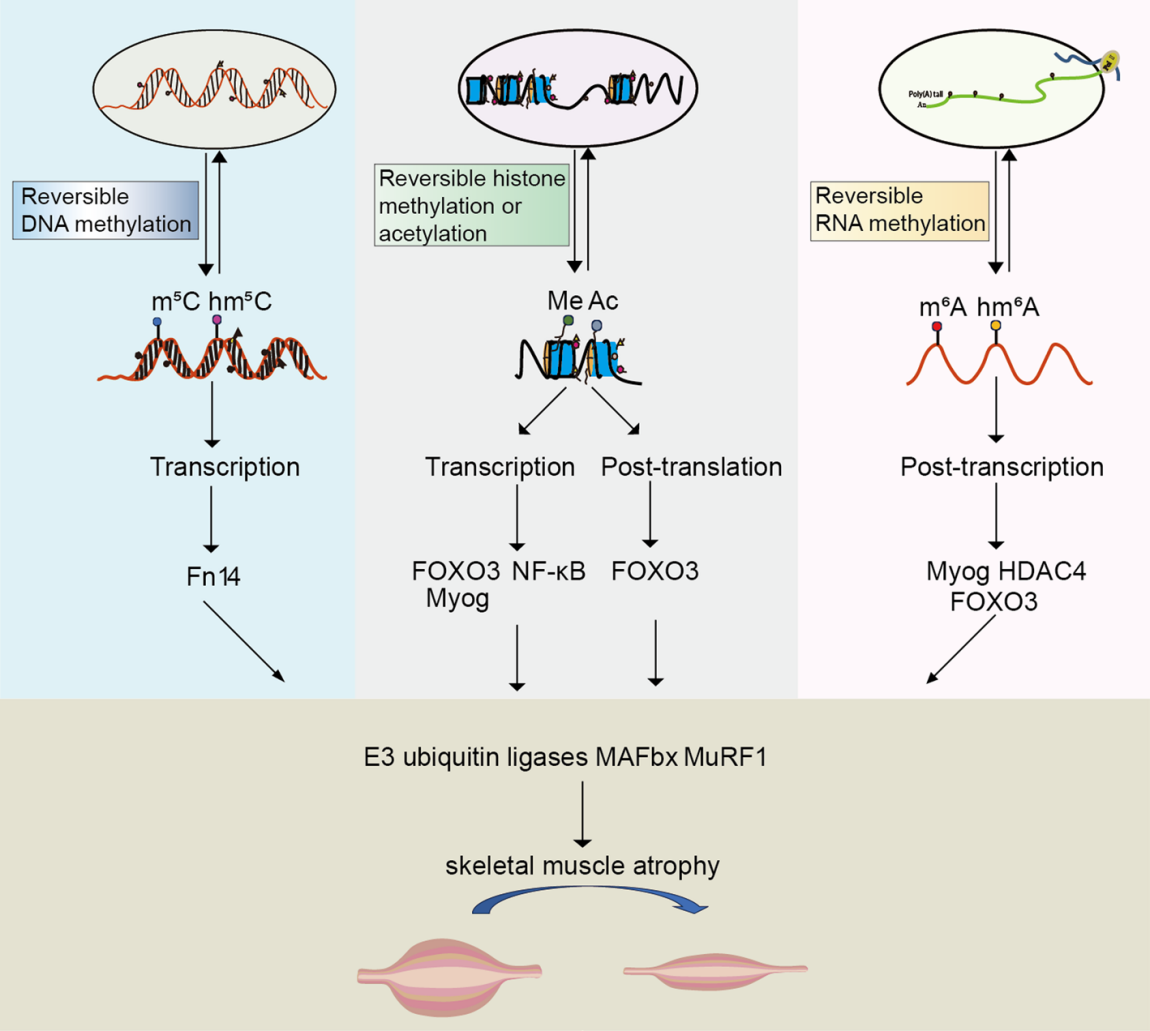
Epigenetic control of skeletal muscle atrophy. Epigenetic modifications regulate the expression of transcription factors such as FoxO3 at the transcriptional, post-transcriptional, and post-translational levels, which in turn activate the E3 ubiquitin ligases MuRF1 and MAFbx, ultimately leading to skeletal muscle atrophy

Beyond influencing genes involved in proliferation and differentiation, Dnmt3a directly modulates the transcription of genes associated with muscle atrophy. The receptor for TNF-like weak inducer of apoptosis (TWEAK), *fibroblast growth factor-inducible receptor 14* (*Fn14*), experiences a significant upregulation during denervation-induced muscle atrophy. This upregulation activates the transcription factor NF-κB, leading to increased expression of *MuRF1* [53] (Fig. 2 and Table 3). Subsequent research has revealed that denervation-induced muscle atrophy results in the hypomethylation of specific CpG sites within the *Fn14* promoter. Overexpression of *Dnmt3a* leads to increased methylation at these sites, suppression of *Fn14* expression, and mitigation of denervation-induced muscle atrophy [54]. Moreover, the reduction in *Dnmt3a* expression observed during denervation-induced atrophy aligns with the hypomethylation of various atrogenes [48]. Consequently, it is suggested that *Dnmt3a* could be a key regulator in the upregulation of atrophy gene expression across different models of skeletal muscle atrophy. This hypothesis was examined in a study utilizing a diabetes mellitus (DM) mouse model of skeletal muscle atrophy. The study demonstrated that continuous administration of Dnmt3a to the tibialis anterior muscle significantly counteracted streptozotocin (STZ)-induced DM muscle atrophy and enhanced myotube formation in C2C12 myoblasts. Crucially, *Dnmt3a* overexpression also reinstated the expression levels of atrophy genes such as *MAFbx*, *MuRF1*, *MyoD*, and *Myog*, both in vivo and in vitro [55].

The expression patterns of DNA methylation writers exhibit contrasting trends in skeletal muscle atrophy models driven by genetic factors versus those induced by denervation and aging. In ALS mice, both 5mC levels and the expression of *Dnmt1* and *Dnmt3a* were found to be increased within the spinal cord and skeletal muscle [56]. Notably, the use of DNMT inhibitors has been demonstrated to significantly alleviate muscle wasting symptoms and enhance motor functions, including body weight and grip strength, in mice suffering from inherited forms of amyotrophy [56, 57]. Further investigations across various animal models of ALS have revealed that Dnmt3a is localized to the outer mitochondrial membrane in muscle fibers. A decrease in mitochondrial *Dnmt3a* expression, alongside an increase in 5mC levels, is associated with mitochondrial loss in skeletal muscle [58]. These findings underscore the dynamic changes in DNA methylation during muscle atrophy due to different causative factors, highlighting the necessity for distinct studies on the expression of methylation writers.

While the expression of *Dnmt1* and *Dnmt3b* remains relatively stable during muscle atrophy, implying a lack of significant change, this steadiness should not be interpreted as an absence of functionality [59]. They are implicated in important processes such as the differentiation of skeletal muscle stem cells and the switching between muscle types. There is a pressing need for further research to elucidate their specific roles and underlying mechanisms associated with skeletal muscle atrophy.

Contradictory reports exist regarding the viability of *Tet1* KO mice. While some studies report that *Tet1* KO results in embryonic lethality [60], others have observed that these mice are viable and fertile, though a proportion of the mutant offspring may exhibit reduced size [61, 62]. Gene expression analysis has identified hypermethylation and altered expression in the promoter regions of genes crucial for skeletal muscle development and contraction. *Tet2* KO mice demonstrate a muscle atrophy phenotype, characterized by a marked decrease in body weight and a reduction in both size and cross-sectional area of the tibialis anterior muscle fibers at ages 2 and 8 weeks [63]. Research by Wang Hongye et al. revealed that *Tet2* deficiency compromises the regenerative ability and myotube fusion in skeletal muscle [64]. Strategies aimed at enhancing *Tet2* stability or ensuring its elevated expression have been shown to reduce the effects of denervation-induced muscle atrophy and improve skeletal muscle function in the context of obesity [65, 66]. Further investigation has shown that *Tet2* deficiency disrupts the proliferation and differentiation of SCs, a process mediated by the calcium signaling gene *Slc8a3* and *Myog*, a critical regulator of myogenesis [63, 64].

In differentiated myotubes cultured in vitro, an increase in the expression of *Tet1* and *Tet2* has been observed, accompanied by a significantly higher level of 5hmC compared with that in undifferentiated C2C12 myoblasts. The reduction of *Tet2* through knockdown experiments has been shown to alter the methylation status and expression of genes critical to myogenesis, thereby impairing the differentiation process of myoblasts [67]. Further investigations have revealed that the differentiation of C2C12 cells is influenced by the phosphorylation of Tet2, a process regulated by AMPK [68]. However, the question of whether the phosphorylation of Tet2 is essential for the proliferation and differentiation of muscle SCs in vivo remains unanswered.

During the regeneration process following muscle atrophy, the expression levels of *Tet1*, *Tet2*, and *Tet3* are all elevated. However, only the suppression of *Tet2* expression significantly impacts myoblast differentiation [64]. This observation indicates that, despite their shared function as deacetylases, each Tet enzyme plays distinct roles in the context of skeletal muscle atrophy and regeneration. Supporting this notion, the knockdown of the methyltransferase *Dnmt3a* and the demethylase *Tet2* exhibits similar effects on muscle SCs. Therefore, DNA methylation regulators can influence skeletal muscle atrophy not through broad changes in genomic methylation but rather through specific regulatory networks and targets, indicating the need for further investigation into their precise mechanisms of action.

### Histone modifications

#### Histone modifications and regulators

DNA is wrapped around an octamer consisting of four core histones: H2A, H2B, H3, and H4. Modifications to these histones are critical in modulating chromatin accessibility, which in turn influences the initiation or repression of gene expression. A vast array of histone post-translational modifications (PTMs) has been identified, encompassing acylation (such as acetylation, benzoylation, butyrylation, crotonylation, glutarylation, lactylation), ADP-ribosylation, dopamine acylation, glycosylation, methylation, phosphorylation, serotonylation, sumoylation, and ubiquitination [69–74]. These modifications, located on the histone tails and within their globular core domains, play direct or indirect roles in the regulation of gene expression, DNA replication, and various other cellular functions by altering the structure of chromatin and the dynamics of protein interactions [75].

Histone acetylation was the first discovered form of histone acyl modification, and it is catalyzed by histone acetyltransferases (HATs). These enzymes catalyze the transfer of acetyl groups from acetyl coenzyme A to histones, forming ε-*N*-acetyl lysine. This addition of acetyl groups reduces the positive charge on histone proteins, weakening the interaction between histones and DNA and consequently leading to the relaxation of chromosomal DNA [76]. The process of histone acetylation enhances DNA accessibility, thereby facilitating the binding of transcription factors and RNA polymerases to specific regions of the chromatin. This facilitates the initiation of transcription, significantly altering chromatin structure and gene expression [77]. HATs are categorized into three main families: the Gcn5-associated *N*-acetyltransferase (GNAT) superfamily, which includes GCN5/KAT2A, PCAF/KAT2B, Ada, and SGAG; the p300/CBP family, which encompasses p300/KAT3B, and CBP/KAT3A; and the MYST family, which consists of members such as Tip60/KAT5, MOZ/KAT6A, MORF/KAT6B, HBO1/KAT7, MOF/KAT8, SAS2, and SAS3 [78, 79] (Table 2). Moreover, certain transcription factors, notably TFIIIC (a universal transcription factor for RNA polymerase III) and CLOCK (an epigenetic regulator of circadian rhythms in skeletal muscle) are recognized for their roles in histone acetylation [80, 81].

**Table 2:** List of histone modification regulators

Histone methylation	SUV39	SUV39H1(KMT1A), SUV39H2(KMT1B), G9a(EHMT2, KMT1C), GLP(EHMT1, KMT1D)	
	MLL	MLL1(KMT2A), MLL2(KMT2D), MLL3(KMT2C), MLL4(KMT2B), MLL5(KMT2E), MLL6(KMT2F), UTX(KDM6A)	
	EZH	EZH1, EZH2, EZH3	
	SMYD	SMYD1, SMYD2, SMYD3, SMYD4	
	PRDM	PRDM1(Blimp-1), PRDM2(RIZ1), PRDM4(PFM1), PRDM5, PRDM9	
	DOT1L	DOT1L	
	PRMT	PRMT1 ~ 11	
Histone demethylation	KDM	KDM1A(LSD1), KDM2A(FBXL11), KDM2B(FBXL10), KDM3A, KDM3B, KDM3C, KDM4A, KDM4B, KDM4C, KDM4D, KDM5A, KDM5B, KDM5C, KDM5D, KDM6A(UTX), KDM6B(JMJD3), KDM7A(PHF8), KDM7B(PHF2), KDM8(JMJD5), KDM9A, KDM9B	
	JMJC	JMJD1A(JHDM2A, KDM3A), JMJD1B(JHDM2B, KDM3B), JMJD2A, JMJD2B, JMJD2C, JMJD2D, JMJD3(KDM6B, UTX), JMJD3L(KDM6A-like, UTY-like), JMJD6, PHF8	
Histone acetylation	HAT	GNAT	GCN5(KAT2A), PCAF(KAT2B), Ada, SGAG
		MYST	Tip60(KAT5), MOZ(KAT6A), MORF (KAT6B), HBO1(KAT7), MOF(KAT8), SAS2, SAS3, ESA1, Nua4(Esa1), MSL
		TAF250	TAF_II_250
		CBP/p300	p300(KAT3B), CBP(KAT3A)
Histone deacetylation	HDAC	I	HDAC1, HDAC2, HDAC3, HDAC8
		IIa	HDAC4, HDAC5, HDAC7, HDAC9
		IIb	HDAC6, HDAC10
		III	SIRT1, SIRT2, SIRT3, SIRT4, SIRT5, SIRT6, SIRT7
		IV	HDAC11

Histone deacetylase (HDAC)-mediated deacetylation of histones results in the formation of closed chromatin structures. HDACs remove acetyl groups from histone proteins, thereby restoring their positive charge and enhancing the interaction between histones and DNA. Consequently, the chromatin structure becomes more condensed, which restricts DNA accessibility [77]. Therefore, HDACs are predominantly associated with the repression of transcription [82]. Eighteen HDACs have been identified in mammals and are categorized into four classes: Class I HDACs encompass HDACs 1, 2, 3, and 8; Class II HDACs are further divided into Class IIa, comprising HDACs 4, 5, 7, and 9, and Class IIb, including HDACs 6 and 10; Class III consists of the sirtuin family members SIRT1, 2, 3, 4, 5, 6, and 7; and Class IV is represented by HDAC11 (Table 2).

Histone methylation is a reversible reaction catalyzed by histone methyltransferase (HMT) and histone demethylase (HDM). Typical histone lysine methylation mainly occurs at sites such as H3K4, H3K9, H3K27, H3K36, H3K79, and H4K20, while the common sites for arginine methylation include H3R2, H3R8, H3R17, H3R26, H2AR3, H4R3, etc.[83]. Different lysine sites can generally undergo monomethylation (me1), dimethylation (me2), and trimethylation (me3) modifications. The process of histone methylation is dependent on specific enzymes that are known as histone methylases, which can be classified as histone lysine methylases and histone arginine methylases, depending on the site at which the modification occurs. Histone lysine methyltransferases (KMTs) are further divided into two categories: enzymes containing a SET domain and proteins without a SET domain, such as DOT1L [84]. Currently, more than 50 proteins containing SET structural domains have been identified in mammals, mainly including lysine methylation enzymes such as the SUV39 family, the mixed-lineage leukemia (MLL) family, the enhancer of zeste homolog (EZH) family, the SET and MYND domain-containing (SMYD) family, and the PRDM family [85, 86] (Table 2). It was found that methylation modifications on histones H3K4, H3K36 and H3K79 are enriched on euchromatin and positively correlate with gene expression, while methylation modifications on H3K9, H3K27, and H4K20 are negatively correlated with gene expression and are usually enriched on heterochromatin or facultative heterochromatin [87].

Histone demethylation (HDMs) involves the removal of methyl groups from histone proteins, converting methylated arginine or lysine residues back to their unmethylated form. This process is facilitated by various mechanisms involving distinct enzyme systems and regulatory pathways. HDMs are classified into two primary categories: the lysine-specific demethylase 1 (LSD1) family of demethylases, which resemble polyamine or monoamine oxidases, utilize flavin adenine dinucleotide (FAD) as a cofactor to demethylate mono- and dimethylated lysines, and the dioxygenase class, which resembles hydroxylases, contains the Jumonji C (JMJC) domain, and uses divalent iron ions and α-ketoglutarate to demethylate trimethylated lysines [73, 88, 89]. These demethylation activities play a crucial role in gene expression and cellular functions by modulating chromatin’s open state, enhancing gene accessibility for transcription, and influencing protein–chromatin interactions, thereby governing cellular processes.

#### Histone modifications dynamic in skeletal muscle atrophy

In aged rats, the gastrocnemius muscle shows a decline in global acetylation levels of histones H3K9 and H3K27, as well as a decrease in the trimethylation of H3K9, which may be associated with age-related skeletal muscle atrophy [90]. In contrast, histone acetylation appears to increase in conditions of muscle atrophy induced by hind limb cast immobilization in the soleus and plantaris muscles, as compared with healthy adult rat skeletal muscle [91]. Similarly, an increase in pan-acetylation of histone H3 is observed in skeletal muscle atrophy induced by denervation [92]. Despite these findings, it is premature to assert that the dynamics of histone acetylation are uniformly opposite between aging and other forms of muscle atrophy, partly due to methodological differences, such as the use of different antibodies (pan-acetyl-lysine antibody for unloading and denervation studies versus acetylated histone H3 (K9, K27) antibody for aging studies), and the examination of different muscle types. The complexities inherent in histone modifications, coupled with the limitations of available antibodies, currently preclude a clear understanding of how methylation and acetylation alterations contribute to skeletal muscle atrophy across different conditions. Although the use of transposases to assess chromatin’s open state could shed light on the dynamics of histone modification, this approach has yet to be widely applied in this context.

Muscle types exhibit distinct patterns of atrophy, a process that may be associated with histone modifications. Ramachandran et al. used chromatin immunoprecipitation sequencing for H3K4me2 and H3K27ac, along with transposase-accessible chromatin profiling, to generate epigenetic profiles for four distinct muscle groups [93]. Their findings indicate that each muscle group possesses unique *cis*-regulatory networks characterized by specific super-enhancers. Further research compared the epigenetic landscapes of the plantaris (a fast-twitch muscle) and the soleus (a slow-twitch muscle) in adult rats, using antibodies against H3K4me3 and pan-acetyl H3 [92]. This comparison revealed that genes in fast-twitch muscles respond more dynamically to physiological stimuli than those in slow-twitch muscles, a response linked to enhanced H3K4me3 and H3 acetylation. Conversely, the expression of functional genes in slow-twitch muscles seems less reliant on these histone modifications [92].

Endurance exercise can decrease the risk of muscle atrophy. Research indicates that just 4 weeks of running training can initiate histone turnover in the skeletal muscle fibers of mice, facilitating nucleosome relaxation and improving the muscle’s genetic response to exercise [94]. Following 6 weeks of endurance training, significant alterations in H3K4me1 and H3K27ac were observed in human skeletal muscle, with the H3K27ac mark being a crucial indicator of enhancer activity that influences the expression of numerous functional genes [95]. Furthermore, acute treadmill exercise in flounder muscle leads to increased histone H3 acetylation at the first exon of the peroxisome *proliferator-activated receptor gamma coactivator 1-alpha* (*PGC-1α*) gene, thereby stimulating *PGC-1α* expression [96]. PGC-1α plays a vital role in enhancing mitochondrial oxidative metabolism in skeletal muscle and elevates the proportion of oxidative muscle fibers [97]. In addition, the expression of *PGC-1α* has been shown to decrease the level of *FOXO3*, which regulates the expression of the downstream ubiquitin ligases *MuRF1* and *MAFbx*, contributing to the prevention of muscle atrophy [98].

Myofibers, the smallest functional units of skeletal muscle, are challenging to analyze due to the presence of hundreds of nuclei within each fiber [99]. Traditional bulk RNA sequencing, proteomics, and epigenomics aggregate signals from various myofibers and cell types, thus failing to accurately reflect the status of individual myofibers. Consequently, single-cell or single-nucleus sequencing methods are also inadequate for detailed myofiber analysis. To address this challenge, Soleimani et al. developed specific transcriptome and epigenome sequencing methods for individual muscle fibers [100, 101]. Through this innovative approach, the chromatin accessibility of single muscle fibers in both normal and CTX-damaged extensor digitorum longus (EDL) muscles was examined. The findings revealed that differences in chromatin accessibility between damaged and undamaged muscle fibers predominantly occur in the intron/distal intergenic region, also known as the enhancer region [25]. Notably, the ATAC peaks identified in most individual EDL fibers overlapped with H3K27ac peaks from whole EDL muscle, as reported by Ramachandran et al., indicating that histone acetylation mediates enhancer activation following muscle fiber injury [25, 93].

#### The roles of histone methylation regulators in skeletal muscle atrophy

Histone lysine methyltransferases are classified into two main categories: those with SET structural domains, including the SUV39, MLL, EZH, SMYD, and PRDM families, and the non-SET domain protein DOT1L. Members of the SUV39 family are known to inhibit the myogenic differentiation of muscle stem cells following skeletal muscle atrophy, primarily by modulating *MyoD* expression. However, research on skeletal muscle fibers in this context is scarce. In vitro experiments have demonstrated that SUV39H1 targets the *MyoD* promoter region, where its persistent methylation results in *MyoD* gene silencing and the inhibition of myogenic differentiation. This effect can be reversed by treating with *SUV39H1* siRNA [102]. Further investigations have shown that p38α MAPK can disrupt the binding between MyoD and KMT1A (SUV39H1) by phosphorylating KMT1A, leading to a transition of the *MyoD* promoter from the transcriptionally repressive state marked by H3K9me3 to an active state indicated by H3K9 acetylation, thus promoting myogenic gene activation and differentiation [103]. G9a and GLP, which are highly homologous, account for the majority of the mono- and dimethylation of H3K9 in euchromatin [104]. In vivo experiments have revealed that the deletion of G9a and GLP in mice leads to embryonic lethality and a significant decrease in H3K9 mono- and dimethylation [105, 106]. Conditional KO mice lacking *GLP* exhibit developmental delays, hypotonia, and other abnormalities [107]. Similar to the roles of SUV39H1 and SUV39H2, G9a and GLP repress myogenic differentiation by negatively regulating the transcriptional activity of *MyoD*. Additionally, PRDM16, from the PRDM family of histone methyltransferases, interacts with G9a and GLP to regulate the deposition of H3K9me2 at the nuclear periphery, consequently silencing the key myogenic differentiation gene *MyoD* [108]. Despite these functions, studies have indicated that G9a might not be essential for muscle development and regeneration in vivo. Conditional KO mice for *G9a* exhibit a significant reduction in total H3K9me2 levels but do not display any noticeable developmental abnormalities in muscle size and appearance [109], suggesting the possibility that *G9a* and *GLP* form heterodimers, with *GLP* potentially compensating for the absence of *G9a* in vivo.

The EZH family, comprising Polycomb proteins EZH1 and EZH2, is another group of histone methyltransferases involved in regulating the transcription of muscle-specific genes. Both EZH1 and EZH2 possess a conserved SET structural domain, enabling them to act as methyltransferases targeting H3K27 [110]. Although EZH1 and EZH2 are paralogs, their functions diverge significantly. Following skeletal muscle injury, muscle stem cells are activated from their quiescent state, initiating muscle regeneration. Swarnali Acharyya et al. demonstrated that, in the dystrophic muscle environment of the DMD model, a high concentration of inflammatory mediators such as TNF-α acts as stimulants for the transcription factor NF-κB, thus activating the NF-κB signaling pathway and promoting muscle degeneration. Further elucidation of the underlying mechanism revealed that the increased levels of TNFα and NF-κB facilitate the recruitment of EZH2 and Dnmt3b to the promoter region of the *Notch-1* gene, which leads to the epigenetic silencing of *Notch-1*, consequently impairing the regenerative capacity of SCs [111, 112]. Silvia Consalvi et al. demonstrated that the E3 ubiquitin ligase Praja1 mediates the degradation of EZH2 following p38α activation, facilitating skeletal myogenesis and highlighting EZH2’s involvement in skeletal muscle regeneration [113]. Apart from this pathway, inflammation has been identified as a factor that activates NF-κB, which in turn targets MyoD, thus inhibiting myogenesis [112]. In the absence of *EZH1*, muscle stem cells spontaneously exit their quiescent state. With repeated muscle injuries, the muscle stem cell pool is progressively depleted, leading to compromised muscle regeneration capabilities. EZH1 plays a crucial role in sustaining the expression of Notch signaling pathway genes in SCs, preventing their premature activation [114].

MLL1, which is upregulated in denervation-induced skeletal muscle atrophy, has been shown to activate the E3 ubiquitin ligases *MuRF1* and *MAFbx*, along with the FOXO3a and NF-κB atrophy pathways. The specific mechanisms through which MLL1 influences skeletal muscle atrophy and its exact relationship with these atrophy pathways remain to be elucidated [115]. Similar to the role of the SUV39 family, MLL1 and its related family members, MLL2, MLL3, and MLL5, could be important in the self-renewal of SCs by regulating the expression of key MRFs such as *MyoD*, *Pax7*, or *Myf5* [116, 117]. In vivo studies indicated that MLL1 and MLL2 contribute to the inhibition of *Pax7* and *MyoD* expression by silencing the HIRA chaperone specific to histone H3.3, adversely affecting the regeneration and self-renewal capabilities of muscle stem cells and leading to decreased muscle cross-sectional area [118]. Notably, conditional KO of *MLL1* in Pax7-positive muscle SCs in mice resulted in significant reductions in muscle mass, cross-sectional area, and muscle fiber diameter, further supporting this observation. However, it has been proposed that MLL2 might not be essential for activating *Pax7* expression in SCs and myoblasts [116]. Pax7 interacts with MLL1 and recruits it to the *Myf5* promoter to maintain H3K4me3 abundance and is required for muscle stem cell proliferation and muscle repair [116, 119]. *MRF4/Myf6* also plays an important role in muscle differentiation, but whether its expression is regulated by epigenetic mechanisms remains elusive [120]. Additional findings reveal that MLL2 and MLL3 can be recruited to the *Myog* promoter region in nucleosomes via the p38 MAPK signaling in C2C12 cells [121]. Furthermore, skeletal muscle-specific KO of *MLL4* in mice has been associated with a downregulation of slow oxidative myofiber gene programs, a decrease in type I myofibers, and diminished mitochondrial respiration, resulting in lowered muscle fatty acid utilization and reduced exercise endurance [122].

The SMYD family, comprising three members, plays a crucial role in muscle integrity, with their deletion leading to muscle atrophy, compromised myofibrillar integrity, and enhanced protein degradation [123, 124]. Conditional KO studies of *SMYD1* in mice and zebrafish have shown a decrease in muscle mass and anomalies in myofibrillar structure [125]. SMYD2’s glutathionylation results in the disintegration of myofibrillar integrity and the degradation of sarcomeric proteins through the action of MMP-2 and calpain-1. This process occurs as SMYD2, upon glutathionylation or oxidation at Cys13, fails to interact with Hsp90 and the N2A domain of nebulin, compromising its sarcomeric stabilizing function [126]. In a mouse model of dexamethasone-induced skeletal muscle atrophy, the methyltransferase SMYD3 was found to exacerbate atrophy by upregulating genes associated with muscle wasting, such as myostatin and the c-Met genes [124]. SMYD3 specifically targets the regulatory regions of the myostatin and c-Met genes, facilitating the recruitment of the bromodomain-containing protein BRD4 through protein–protein interactions. This recruitment allows for the chromatin engagement of the pause-release factor p-TEFb and the elongation of Ser2-phosphorylated RNA polymerase II, ultimately diminishing the transcription of myostatin and c-Met and influencing glucocorticoid-induced myotube atrophy [124].

The DOT1L family, while not extensively studied in the context of skeletal muscle atrophy, has shown pivotal implications in embryonic mice where its absence was associated with a greater risk of mortality [127]. Additionally, in patients suffering from chronic obstructive pulmonary disease (COPD), a notable downregulation of the histone methyltransferase *DOT1L* has been observed in the vastus lateralis muscle. In vitro analyses further reveal that *DOT1L* gene suppression in human skeletal muscle SCs correlated significantly with increased cellular arrest and aging markers, specifically *p21WAF1*/*Cip1*/*CDKN1A* [128].

In addition to protein lysine methyltransferases, protein arginine methyltransferases (PRMTs) have been found to be important regulators of skeletal muscle metabolism and regeneration, and their roles in skeletal muscle have been summarized in the literature [129–137] (Table 2). PRMT family members primarily contribute to the regulation of skeletal muscle atrophy through mechanisms involving autophagy or degradation pathways mediated by E3 ubiquitin ligase, as well as pathways supporting muscle stem cell regeneration. These pathways include the regulation of *MyoD* expression, enhancement of myogenic differentiation, and facilitation of muscle regeneration [130, 138–140]. Specifically, mice with a skeletal muscle-specific KO of *PRMT1* exhibited decreased body weight and muscle mass and elevated levels of *FOXO3*, the muscle-specific ubiquitin ligase *MuRF1*, and the autophagy marker *LC3-II* [135]. Further research indicates that PRMT1 modulates autophagy via the PRMT6–FOXO3 pathway and protein degradation mechanisms. During denervation, PRMT1 engages with AMPK and PGC-1α to establish the PRMT1–AMPK–PGC-1α signaling axis, potentially active in muscle remodeling scenarios [141]. Similarly, PRMT7 appears to influence *PGC-1α* expression through interaction with its upstream regulator p38 and activating transcription factor 2 [142, 143]. Conversely, CARM1 (alias of TRMT4) is integral to autophagy regulation. It modulates autophagy through the AMPK–SKP2–CARM1 signaling pathway. Under conditions of glucose abundance, nuclear CARM1 is degraded by the SKP2-containing E3 ubiquitin ligase SCF (SKP1-cullin1-F-box protein). In addition, glucose deprivation leads to nuclear accumulation of AMPK, which phosphorylates FOXO3, enhancing its transcriptional activity. FOXO3, in turn, transcriptionally represses SKP2, resulting in the stabilization of CARM1 protein and an increase in histone H3R17 dimethylation, triggering autophagy [144]. These findings indicate that PRMT family member expression induction during denervation might serve as a compensatory mechanism to restrict skeletal muscle atrophy. Nonetheless, the potential of their overexpression or continuous activation in preventing muscle atrophy and the involvement of common signaling pathways, such as those associated with PGC-1α or FOXO3, remains to be investigated.

HDMs, including members of the KDM and JMJC families (Table 2), are recognized for their role in the epigenetic regulation of muscle stem cells [88, 89, 145]. Similar to histone methyltransferases, HDMs primarily influence myogenic differentiation via the regulation of *MyoD* expression. However, certain studies have also identified their role in the atrophy mechanisms within skeletal muscle fibers. The depletion of *LSD1* in skeletal muscle fibers amplifies glucocorticoid-induced atrophy in fast-twitch muscle fibers by diminishing the nuclear retention of the anti-autophagy transcription factor Foxk1, achieved through the inhibition of the Akt–mTORC1 axis [146]. This reduction in Foxk1 allows for the replacement of its target genes with those of *FOXO3*, enhancing the accessibility of atrophy-associated genes within muscle cells [147, 148]. Conversely, in the presence of abundant nutrients, the Akt–mTORC1 pathway fosters the nuclear presence of Foxk1, thereby suppressing autophagy and other catabolic processes [149]. Research into hypoxic conditions has revealed that the signaling cascade involving mechanistic target of rapamycin (mTOR), NF-κB, and TGF-β pathways can elevate the expression of *hypoxia-inducible transcription factor-1α* (*HIF-1α*). This, in turn, stimulates the expression of *KDM3A*, *KDM4B*, *KDM4C*, and *KDM6B*, facilitating gene expression through demethylation regulation [150, 151]. Furthermore, conditions such as oxidative stress, impaired energy metabolism, and inflammation associated with skeletal muscle atrophy are known to activate the mTOR, NF-κB, and TGF-β signaling pathways. Nevertheless, the specific regulatory interactions between HDMs and these pathways remain largely unexplored. While some HDMs may interact with the oxidative stress-responsive transcription factor FOXO [152], the exact signaling pathways they regulate and their impacts on muscle fibers in models of skeletal muscle atrophy warrant further investigation.

#### The mechanism of histone acetylation regulators controlling skeletal muscle atrophy

Research into protein acetylation is well documented, with numerous publications systematically detailing the influence of different histone acetylation regulators on skeletal muscle atrophy [79, 96, 171]. In this section, we provide insights into how several key regulators act on common myasthenic pathways leading to muscle atrophy.

P300/CBP-specific KOs in the skeletal muscle of transgenic mice exhibit no alterations in muscle structure, mass, fiber type, or locomotor activity [172]. A combined KO of *P300* and *CBP* within skeletal muscle does not impact muscle mass or structure but significantly compromises muscle strength and contractility [173]. This finding does not suggest that *P300/CBP* primarily influences skeletal muscle physiology over structure and mass. In an in vitro muscle atrophy model, *P300/CBP* was found to enhance the acetylation levels of transcription factors critical for muscle mass regulation, including C/EBP, FOXO, and NF-κB/p65. Inhibition of P300/CBP-mediated acetylation of these transcription factors led to a decrease in the expression of the ubiquitin ligase *MuRF1*, subsequently mitigating myotube atrophy [174]. The function of P300/CBP in the atrophy of skeletal muscle primarily involves the modulation of FOXO family transcription factors [175]. CBP/p300-mediated non-histone acetylation facilitates the formation of a cysteine-thiol disulfide-dependent complex with FOXO transcription factors, leading to a negative regulation of FOXO activity at the post-translational level [176, 177]. Additionally, CBP/p300-mediated histone acetylation suppresses *FOXO* expression at the transcriptional level [164]. FOXO transcription factors target the promoter of *MAFbx*, triggering rapid transcription of *MAFbx* and resulting in significant atrophy of myotubes and myofibers [178] (Fig. 2 and Table 3). Inhibiting the transcriptional activity of *FOXO* or reducing its expression substantially mitigates skeletal muscle atrophy [179]. The supplementation of P300 in disuse atrophic skeletal muscle has been shown to decrease *FOXO* and *MAFbx* expression [164]. Recent studies in various models have broadened our understanding of the molecular mechanisms by which CBP/p300 contributes to skeletal muscle atrophy. In diabetes-induced atrophy, high fat and insulin resistance were shown to promote CBP/p300 phosphorylation. This modification enhances its acetyltransferase activity, subsequently activating autophagic processes and leading to atrophy-associated morphological and molecular alterations [180]. Further mechanistic insights have been gained from examining skeletal muscle atrophy in cancer cachexia, where it has been reported that cancer cells activate Toll-like receptor 4 in skeletal muscle, which then phosphorylates CBP/p300 via the p38β–MAPK pathway [181]. Within myotubes exposed to a pro-oncogenic environment, phosphorylated CBP/p300 increases the acetylation of the transcription factor C/EBPβ at the Lys39 residue, thereby activating it [162]. This activated C/EBPβ then binds to *cis*-elements in the promoter region of the *MuRF1* and *MAFbx* gene independently of FOXO, leading to an upregulation of *MuRF1* and *MAFbx* expression and consequent skeletal muscle atrophy [163].

**Table 3:** Functional epigenetic regulators of skeletal muscle atrophy and their targets

Calss	Epigenetic regulators	Targets
DNA modifications	DNMT3A	*Gdf5*, *p57Kip2* [50, 52], *Fn14* [53]
	Dnmt-3B	*Notch-1* [111, 112]
	TET2	*Slc8a3*, *MyoG* [63, 64]
RNA modifications	METTL3	*NPNT* [153], *MyoD* [154], *YTHDF1/2* [155, 156]
	ALKBH5	*Hdac4* [157], *FoxO* [157, 158]
	FTO	*FoxO* [158], *mTOR*, *PGC-1α* [159], *TGFβ1* [160]
Histone modifications	SUV39H1	*MyoD* [102], *p38α* [103]
	G9a, GLP	*MyoD*, *PRDM16* [108]
	EZH1	*Notch* [114]
	EZH2	*Notch-1* [111], *Praja1*, *p38α* [113]
	MLL1	*HIRA* [118], *MyoD*, *PAX7*, *Myf5* [116, 117]
	MLL2	*HIRA* [118], *Ash2L* [121], *MyoD*, *PAX7*, *Myf5* [116, 117]
	MLL3	*Ash2L* [121]**,** *MyoD*, *PAX7*, *Myf5* [116, 117]
	MLL5	*PAX7*, *Myf5* [117]
	SMYD2	*Hsp90*, *N2A* [126]
	SMYD3	*Myostatin*, *c-Met*, *BRD4* [124]
	PRMT1	*FoxO3* [135], *AMPK*, *PGC-1α* [141]
	PRMT4	*Atrogin-1*, *MuRF1*, *PGC-1α* [161], *SKP2* [144], *FoxO3* [136]
	PRMT7	*p38* [142, 143]
	LSD1	*Foxk1* [148]
	KDM3A, KDM4B, KDM4C, KDM6B, KDM2B, KDM5B	*HIF-1α* [150, 151]
	P300/CBP	*C/EBPβ* [162], *Atrogin1/MAFbx* [163], *FoxO* [164]
	HDAC1	*FoxO* [165], *KLF5* [166, 167], *C/EBPβ* [168]
	HDAC4	*MyoG* [169], *AP1* [170]

HDAC1, functioning oppositely to CBP/p300, similarly contributes to the induction of skeletal muscle atrophy. During skeletal muscle atrophy prompted by nutritional deprivation, the transcriptional activation of *FOXO* is contingent upon histone deacetylation. Changes in *HDAC1* expression alone can activate *FOXO*, leading to the upregulation of *MAFbx* and the promotion of muscle wasting and fiber atrophy [165]. Furthermore, HDAC1 plays a role in the deacetylation of the zinc finger transcription factor Krüppel-like factor 5 (KLF5), which negatively impacts its protein level expression [166, 167]. KLF5 is a mediator in the early phase of skeletal muscle atrophy, with its muscle-specific deletion markedly mitigating atrophy induced by mechanical unloading. The mechanism underlying this involves KLF5’s interaction with FOXO1 and its co-regulatory effect on the transcriptional activity of *MuRF1* and *MAFbx* [182]. Intriguingly, the removal of KLF5 also led to decreased H3K27ac levels at the *MuRF1* promoter and enhancer regions [182]. Additionally, autophagy is targeted by HDAC1. Mice lacking HDAC1 and HDAC2, specifically in skeletal muscle, exhibited higher mortality rates along with ultrastructural defects, such as sarcomere disruption and mitochondrial abnormalities, yet without overt muscle atrophy [183]. Subsequent research indicated that HDAC1 and HDAC2 influence autophagosome formation, though it remains uncertain whether this effect relies on their deacetylase activity. HDAC1 has also been shown to complex with C/EBPβ, co-regulating downstream pathways including p53, SIRT1, and PGC1α, though their precise roles in skeletal muscle atrophy remain to be elucidated [168].

HDAC4 is one of the first epigenetic factors identified to directly regulate skeletal muscle atrophy. Its expression is upregulated in skeletal muscle atrophy induced by various factors such as denervation, wasting, and aging [184]. Notably, HDAC4 is primarily localized at the neuromuscular junction (NMJ), and upon denervation, it dissociates from this site, leading to a significant induction of its expression and subsequent nuclear accumulation [185], suggesting HDAC4’s increased sensitivity to neurogenic skeletal muscle atrophy. One of the key mechanisms via which HDAC4 mediates muscle atrophy is by inhibiting myogenic protein *Myog*, which acts as a transcription factor and directly targets the promoters of *MuRF1* and *MAFbx*, thereby upregulating their transcriptional expression [169, 186]. The forced expression of *Myog* in skeletal muscles of mice lacking HDAC4 can reverse the muscle atrophy that ensues post-denervation. However, the precise mechanism of how HDAC4 suppresses *Myog* expression—whether through direct acetylation of the *Myog* protein or via transcriptional regulation through histone deacetylation—remains unclear. HDAC4 also targets other downstream molecules in skeletal muscle, particularly under conditions of denervation. For example, the transcription factor AP1 is a significant target, with HDAC4 activating AP1 not through its canonical transcriptional repression function but by deacetylating MAP kinase, thereby activating AP1 via the MAPK pathway [170]. A systematic analysis of the downstream molecules affected by *HDAC4* KO in mouse skeletal muscle revealed that *HDAC4* influences numerous noncoding RNAs and the competitive endogenous RNA (ceRNA) networks they form, within which lncRNAs such as *XR_377582.2* and *ENSMUST00000143649* have been identified as potentially crucial in regulating muscle atrophy in relation to HDAC4 [187]. HDAC4’s role in skeletal muscle atrophy is not initiated independently; rather, it is preceded by the activity of signaling pathways such as PKB/Akt and mTORC1 following nerve injury. These pathways promote the nuclear import of HDAC4, which in turn facilitates the expression changes in numerous genes associated with muscle atrophy [188].

It is worth noting that HDAC4 also plays an important role in the genetic neuromuscular disease ALS. In the skeletal muscle of ALS patients and mouse models, the level of HDAC4 has been reported to be much higher than in normal controls [189, 190]. Using a genetic strategy to knock out HDAC4 in the skeletal muscle of SOD1 mice was found to result in early onset of ALS, manifested by weight loss, muscle denervation and atrophy, and impaired muscle performance [191]. In contrast to nonhereditary muscle atrophy, catabolic pathways such as the ubiquitin–proteasome are not activated, and the specific pathways leading to atrophy remain unclear.

Several HDAC inhibitors (HDACIs), such as trichostatin A (TSA) and butyrate, have been utilized to treat skeletal muscle atrophy, effectively inhibiting the expression of *MuRF1* and *MAFbx* and alleviating skeletal muscle atrophy in a mouse model [192, 193]. However, the application of HDACIs in treating hereditary skeletal muscular atrophy, including ALS, remains challenging. In diseases such as ALS, not only the skeletal muscle but also motor neurons and NMJs are involved, leading to different sensitivities to HDACIs. For instance, the intraspinal injection of the HDACI sodium valproate effectively protects motor neuron cell bodies but fails to prevent the degeneration of NMJs [194].

Similarly, while butyrate has shown efficacy in alleviating skeletal muscle atrophy, it has a limited effect on protecting motor neurons against chronic spinal motor neuron death induced by AMPA-mediated excitotoxicity in an ALS model [195]. Thus, more targeted treatment strategies are required for these complex genetic disorders. Additionally, despite the current classification of HDACs and HDACIs, the specific matching of these inhibitors to their appropriate substrates is not fully understood, adding uncertainty to the clinical application of HDACIs.

### RNA modifications

#### RNA modifications and regulators

Similar to DNA, all forms of RNA—including messenger RNA (mRNA), noncoding RNA, ribosomal RNA (rRNA), and transfer RNA (tRNA)—undergo modifications that, while not altering their nucleotide sequence, significantly impact their function and destiny. This phenomenon, known as epitranscriptomics, highlights the role of RNA modifications in gene expression and cellular function. RNA modifications are diverse, with over 150 identified to date. Among these, N^6^-methylation of adenosine (m6A) is the most abundant in eukaryotes, accounting for more than half of all RNA methylation in cells [196, 197]. As the name suggests, this modification involves the addition of a methyl group to the sixth nitrogen atom of adenine. Other well-characterized RNA modifications include N^1^-methyladenine (m1A) modification, 5-methylcytosine (m5C) modification, 3-methylcytosine(m3C) 7-methylguarine (m7G) modification, N^6^, 2′-*O*-dimethyladenine nucleoside (m6Am), pseudouridine (Ψ) modification, etc. [198] (Table 4).

**Table 4:** List of RNA modifications and regulators

RNA methylation	m^6^A	METTL3, METTL14, WTAP, VIRMA, KIAA1429
	m^5^C	RsmB/Yn1022c, NOP2/NOL1, YebU/Trm4, PH1991/NSUN
	m^7^G	RNMT
	m^6^A_m_	2*’-O-*MTase, 2*’-O-*methyladenosine*-N*^*6*^
RNA demethylation	m^6^A	FTO, ALKBH5
	m^5^C	TET
	m^1^A	ALKBH3
	m^6^A_m_	FTO

The m6A modification is the most common form of RNA modification and is widely distributed in various RNAs. Studies have identified a nonuniform distribution of m6A across mRNA, showing a preference for the coding sequence (CDS) and the 3′ untranslated region (3′UTR), particularly in regions proximal to the stop codon. The occurrence of m6A is sequence dependent, predominantly appearing on adenines within the conserved RRACH motif (R = A or G; H = A, C, or U). This motif significantly overlaps with the binding sites of the SRSF splicing factor, suggesting that exons modified by m6A are more likely to be included in mRNA transcripts [199, 200]. Research on other RNA modifications, particularly in tRNAs and rRNAs, is limited due to their scarce presence and the challenges associated with detecting them with high sensitivity. The enzymatic process leading to m6A modification involves a methyltransferase complex that includes methyltransferase-like 3 (METTL3), METTL14, Wilms tumor 1 associated protein (WTAP), and Vir like m6A methyltransferase associated (VIRMA), collectively referred to as the “writers” of m6A. Conversely, the demethylation of m6A is facilitated by enzymes such as alpha-ketoglutarate-dependent dioxygenase Alk B homolog 5 (ALKBH5) and fat mass and obesity-associated protein (FTO), known as “erasers,” which can reverse m6A modifications. The dynamic interplay between writers and erasers contributes to a global modulation of m6A levels. The regulatory impact of m6A modifications on gene expression is mediated through its influence on mRNA stability or protein translation, which is accomplished by recruiting various m6A-binding proteins, or “readers.” Recognized readers include the YTH domain-containing proteins (YTHDF1, YTHDF2, YTHDF3, YTHDC1, and YTHDC2) and members of the heterogeneous nuclear ribonucleoprotein (HNRNP) family (HNRNPA2B1 and HNRNPC). It is proposed that the number of such readers may be considerably higher [201, 202]. The regulatory mechanisms for other RNA methylation modifications also involve distinct sets of writers and erasers, which are detailed in Table 4.

#### RNA modification regulators in skeletal muscle atrophy

RNA modification plays a crucial role in virtually all metabolic processes of RNA, from synthesis to degradation [203], with research confirming that m6A methylation is pivotal in various aspects of skeletal muscle physiology. Existing reviews have comprehensively summarized its significance in skeletal muscle development [204, 205], as well as in other skeletal muscle disorders [206, 207].

The aging of primate skeletal muscle is marked by a decline in *METTL3* expression and a consequent reduction in m6A methylation. Deletion of *METTL3* in myotubes derived from human pluripotent stem cells leads to a significant decrease in both myotube diameter and nuclei count. *NPNT*, a downstream target of *METTL3*, exhibits decreased mRNA and m6A levels in aging myoblast tubes, mirroring the phenotype observed with *METTL3* deletion [153]. Experimental supplementation of *METTL3* in vivo promotes skeletal muscle growth, whereas conditional genetic deletion of *METTL3*, specifically in myofibers, results in spontaneous muscle atrophy. These findings indicate that *METTL3* expression levels are a critical determinant of skeletal muscle mass [208]. Furthermore, *METTL3* influences the expression of numerous skeletal muscle-specific noncoding RNAs (ncRNAs) through m6A modification [209, 210], suggesting that ncRNAs, which play a crucial role in muscle atrophy, may represent one of the mechanisms by which *METTL3* modulates skeletal muscle mass [211].

The promoting effect of *METTL3* deletion on skeletal muscle atrophy may be linked to its impact on muscle SCs. Specific reduction in *METTL3* expression within muscle SCs markedly suppresses the proliferation of these muscle stem cells and impairs muscle regeneration post-atrophy [212]. Conversely, overexpressing *METTL3* significantly enhances both the proliferation and myogenic differentiation of C2C12 myoblasts [156]. In proliferating muscle SCs, *METTL3* is essential for sustaining m6A levels in the 5′ untranslated region (5′UTR) of the myogenic transcription factor *MyoD* mRNA. *MyoD* mRNA levels are notably decreased following *METTL3* knockdown [154]. Moreover, *METTL3* plays a pivotal role in managing the transition of muscle SCs/myoblasts from a proliferative to a differentiated state. Suppressing METTL3 expression leads to premature differentiation of myoblasts and boosts the engraftment of muscle SCs after their initial transplantation [213].

m6A readers work in conjunction with *METTL3* in an m6A-dependent manner. Specifically, *YTHDF1/2* influences the mRNA stability of MRFs such as *STK11*, *MyHC*, *MyoD* and *Myog*, and plays a role in their translation [155, 156]. In some cases, these readers directly impact the myoblast function. For instance, during muscle regeneration following acute injury, *YTHDC1* regulates SCs activation and proliferation. SCs lacking *YTHDC1* fail to exit quiescence, and their regenerative capacity is significantly compromised [214, 215]. Mechanistically, *YTHDC1* interacts with m6A-modified mRNAs to influence their alternative splicing, with these mRNAs primarily involved in pathways related to the regulation of actin filament-based processes and PI metabolism [214, 215].

Both our research and findings from another independent team indicate that loss of innervation in skeletal muscle leads to a reduction in global m6A levels, similar to the decline observed in aging skeletal muscle [157, 216], suggesting a reduction in m6A might be a universal characteristic of skeletal muscle atrophy. However, in denervated muscle atrophy, the decrease in m6A is predominantly due to demethylation, marked by the upregulation of the demethylases *FTO* and *ALKBH5*. Specifically, *ALKBH5* increases the stability of *HDAC4* mRNA through demethylation, activating the HDAC4-FOXO3 pathway and resulting in muscle atrophy [157] (Fig. 2 and Table 3). Our research further demonstrates that, during denervated muscle atrophy, m6A modification of several genes associated with the ubiquitin–proteasome pathway is reduced, leading to their elevated expression, indicating that reduced m6A is a contributing factor to the activation of the ubiquitin–proteasome pathway [216]. Furthermore, we also explored m6A’s role in skeletal muscle atrophy through pharmacological interventions. The results indicated that FTO inhibitor *R*-2-hydroxyglutarate (R-2HG) in denervated tibialis anterior muscle increased total m6A levels, ameliorating muscle atrophy, while treatment with 3-deazadenosine (Daa) in healthy tibialis anterior muscle reduced m6A levels, resulting in spontaneous muscle atrophy [216]. Importantly, R-2HG specifically inhibited FTO but not ALKBH5, while Daa did not selectively target m6A regulators. Despite both ALKBH5 and FTO recognizing m6A-modified RNAs, differences in their substrate preferences, cellular localization and reaction products exist [217–219]. These findings imply that the impact of m6A on skeletal muscle atrophy is related to the overall m6A level rather than specific regulatory proteins, which is further illustrated by the synergistic action of FTO and ALKBH5 in activating the FOXO pathway in some contexts [158].

The role of FTO in myoblasts presents a complex picture, challenging the notion of its straightforward impact on overall skeletal muscle atrophy. Research indicates that, while FTO is essential for myogenic differentiation, an excess of FTO does not enhance myotube formation [159]. It was observed that silencing FTO impaired mitochondrial biogenesis and energy production, attributable to the downregulation of the *mTOR* and *PGC-1α* [159]. Further investigation revealed that m6A modification of the 5′UTR of TGFβ1 mRNA facilitates its degradation, with FTO influencing these modifications to hinder myoblast differentiation [160]. Experiments in genetically engineered mice demonstrated that FTO overexpression did not alter skeletal muscle mass [220], whereas FTO deficiency compromised skeletal muscle development [159]. This discrepancy may be elucidated by considering two aspects: firstly, the differentiation of SCs into myotubes might differ between developmental processes and post-injury repair; secondly, m6A modifications may exert varying impacts across different cell types or subtypes, with their collective interactions influencing skeletal muscle atrophy [221, 222].

Research on other RNA modifications in the context of skeletal muscle atrophy remains limited, potentially due to challenges in detecting these modifications with high sensitivity. One study utilizing pigs as a model organism demonstrated a positive correlation between m5C mRNA levels in skeletal muscle and muscle mass, indicating a possible regulatory role of m5C in skeletal muscle atrophy [223]. The lack of functional studies on various RNA-modifying writers, readers, and erasers in human and rodent models hinders further advancement in the biomedical research of skeletal muscle atrophy.

## Epifactors and alternative splicing crosslinks during muscle atrophy

Alternative splicing (AS) and epigenetic regulation are crucial mechanisms that steer gene expression. In certain physiological or pathological contexts, adjacent splice sites on pre-mRNAs compete, resulting in the generation of various splice isoforms. These isoforms can have differing or even opposing functions, significantly expanding the repertoire of functional proteins and enhancing cellular complexity [224]. Pre-mRNAs contain numerous *cis*-regulatory elements that interact with *trans*-acting factors [notably, splicing factors, primarily RNA-binding proteins (RBPs)] to collaboratively determine AS outcomes [225]. Furthermore, AS is modulated by alterations in RNA secondary structure [226, 227]. In patients with skeletal muscle atrophy and corresponding animal models, numerous aberrant AS events have been documented that contribute to muscle atrophy [228–231]. Moreover, significant skeletal muscle atrophy has been both induced and reversed by genetically manipulating RBPs [232–240]. Our research identified that, in a model of denervation-induced atrophy, AS modification of a broad array of structural and functional proteins associated with muscle atrophy aligns with the atrophic phenotype. Remarkably, we observed a substantial increase in several highly conserved exons (HCEs) of *Obscn* following three days of denervation [241]. Similar outcomes have been observed in the skeletal muscles of mice subjected to prolonged microgravity conditions in space [242]. The *Obscn* gene encodes obscurin, a large structural protein critical for myofibril assembly [243]. We propose that HCEs are rich in RNA secondary structures, specifically G-quadruplexes, which may trigger cytotoxicity and consequently lead to skeletal muscle atrophy [241].

**Fig. 3:**
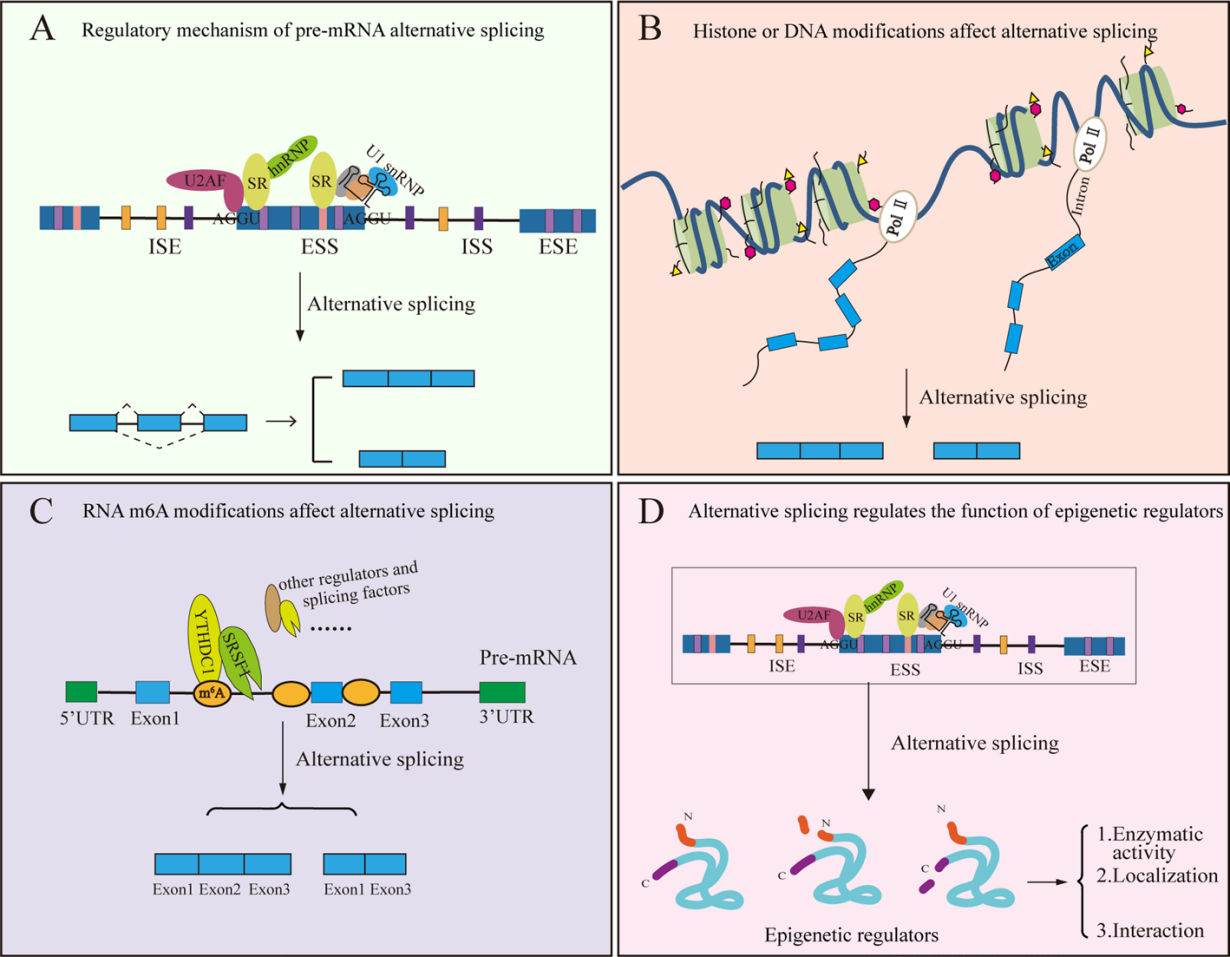
Epigenetic and alternative splicing are intertwined. **A** Regulatory mechanism of pre-mRNA alternative splicing. Splicing regulatory elements such as exonic splicing enhancers (ESEs), exonic splicing silencers (ESSs), intronic splicing enhancers (ISEs), and intronic splicing silencers (ISSs), etc., which are located around the splice site. Splicing factor SR proteins or hnRNPs can bind to them and recruit core splicing elements such as U2AF and U1 snRNP to regulate alternative splicing. **B** Histone or DNA modifications affect alternative splicing. Transcription and splicing occur almost simultaneously in the same space, and various epigenetic modifications can alter the rate of Pol II elongation during transcription, which can affect recognition of the splice site by the spliceosome and, thus, alternative splicing. **C** RNA m6A modifications affect alternative splicing. Many m6A readers are splicing factors themselves, which can regulate numbers of alternative splicing events. m6A regulators are also able to recruit splicing factors to participate in alternative splicing regulation, e.g., YTHDC1 binds to pre-mRNAs in an m6A-dependent manner, and it can recruit the splicing factor SRSF1 to regulate alternative splicing. **D** Alternative splicing regulates the function of epigenetic regulators. The expression of multiple epigenetic regulators is regulated by alternative splicing, which produces full-length isoforms and N-or C-terminally reduced protein products. These truncated isoforms differ in enzymatic activity, cellular localization, and interactions

In skeletal muscle tissue and muscle stem cells, the *Obscn* undergoes DNA methylation, predominantly characterized by hypermethylation [40]. Hypomethylated sites in the untranslated regions (UTRs) might correlate with gene expression, whereas the abundance of hypomethylated sites across exons could relate to the generation of numerous AS variants. Indeed, the processes of epigenetics and AS are intricately linked and jointly influence skeletal muscle atrophy. Epigenetic modifications and their enzymes (writers) can influence AS, while the mechanism of AS itself can prompt epigenetic regulators to produce variant isoforms with distinct functions (Fig. 3). Although we have extensively reviewed the dynamics of epigenetic changes in skeletal muscle atrophy and the roles of epigenetic writers, a direct connection between RNA AS and epigenetic inheritance in skeletal muscle has yet to be established. Below, we outline three hypothetical models that could shed light on the regulatory mechanisms governing skeletal muscle atrophy and regeneration.

### Epigenetic modifications affect AS

Epigenetic modifications, particularly DNA methylation and histone modifications, have a direct, global and significant impact on AS [244]. Genome-wide, DNA methylation can alter the splicing of approximately 20% of alternative exons. Heterochromatin protein 1 (HP1) detects DNA methylation in a location-specific manner and functions as a linker protein, facilitating the recruitment of the splicing factor SRSF3 to methylated sites [245]. Histone modifications, including methylation and acetylation, also affect the recruitment of splicing regulators via chromatin-binding proteins, impacting the splicing outcomes of thousands of genes [246–248]. For instance, the histone modification H3K36me3, recognized by the *MORF-related gene 15* (*MRG15*), influences the splicing of numerous variable exons by recruiting the splicing factor PTBP1 [246]. The removal of the H3K36me3 mark’s writer, SETD2, during skeletal myogenesis triggers simultaneous shifts in gene expression and AS, disrupting myotube formation through metabolic reprogramming [249].

Spliceosome assembly and transcription processes occur almost simultaneously within the same cellular compartments, and epigenetic modifications also affect a large number of AS by altering the rate of transcription elongation [250]. The DNA-binding protein CCCTC-binding factor (CTCF) is involved genome-wide in mediating local RNA polymerase II pauses to promote the inclusion of weakly upstream exons, whereas 5mC expels CTCF, leading to exon skipping. Inhibiting DNMT1 expression helps maintain the slow rate of RNA polymerase II and promotes co-transcriptional spliceosome assembly at weak splice sites [251]. Decreased TET levels can increase 5mC, leading to CTCF expulsion and alternative exon skipping [252]. Acetylation modification of histone H3K9 increases the rate of RNA polymerase II, whereas methylation modification decreases it, dynamically generating two distinct splicing patterns [253]. Numerous histone modifications can affect the speed of RNA polymerase II, as detailed in the literature [254] (Fig. 3B).

### M6A modifications and regulatory recruitment of splicing factors regulate AS

Approximately 10% of the m6A signal is found in variable exons and adjacent introns, indicating a potential relationship between m6A modifications and AS [255]. The presence of m6A at splice sites, co-transcribed with mRNA, facilitates the splicing mechanism, leading to an increase in alternative exon exclusion [256]. Further analysis of existing epitranscriptomic datasets has uncovered the co-localization of G-quadruplex structures and m6A modifications, which are particularly enriched near splice sites [257] and align with the observation that nearly all known m6A regulators influence global AS patterns in an m6A-dependent fashion [258–261]. Among these regulators, YTHDC1 can bind several SRSF family members, recruiting them to m6A sites and thereby influencing a broad spectrum of splicing events [200, 262, 263]. Mice with reduced SRSF1 levels appeared normal at birth but exhibited significant skeletal muscle atrophy and died within 5 days, highlighting SRSF1’s critical role in satellite cell proliferation and the establishment of functional NMJs [264] (Fig. 3C).

Beyond YTHDC1, the m6A reader IGF2BP2 has been implicated in the activation of muscle SCs [265]. In the context of sarcopenia and in orchiectomized mice, downregulation of IGF2BP2 by miRNA Let-7e-5p was observed, which hindered myosin heavy chain development and energy metabolism, contributing to muscle atrophy [266]. However, it is not clear whether the function of IGF2BP2 is related to the AS.

### AS of epigenetic regulators

Functional splice isoforms have been identified for all members of the DNA methyltransferase family [267]. A specific *DNMT1* isoform is present in mouse skeletal muscle, distinct from those in most peripheral tissues. This isoform is truncated at the N-terminus and is specifically expressed in terminally differentiated muscle cells, potentially influencing de novo DNA methylation during the myogenesis process [268]. Numerous *DNMT3b* splice isoforms have been documented in humans and rodents [269–271]. These isoforms serve distinct functions: some alter the subcellular localization of *DNMT3b*, others affect its affinity for DNA, and some function as helper proteins that positively regulate de novo methylation by *DNMT3a* and *DNMT3b*, despite lacking catalytic activity [271–273]. Additionally, the DNA demethylase *Tet1* and its truncated isoforms exhibit different nuclear localizations and target m5C at various positions, leading to altered cytosine modification levels in human and mouse cells [274].

Histone modification regulators frequently undergo AS. For skeletal muscle to regenerate following injury, it is necessary to remove repressive chromatin marks from the promoter of the *Myog*. As myoblasts differentiate into myotubes, the HDM *JMJD2A/KDM4A* transitions to an isoform devoid of the N-terminal demethylase domain (ΔN-JMJD2A), playing a crucial role in the demethylation of H3K9me2 and H3K9me3 at the *Myog* promoter [275] (Fig. 3). Recent research has shown that numerous genes encoding histone-modifying enzymes are subject to AS under specific physiological conditions. For instance, the AS of exon 9 in *HDAC7* significantly affects global histone modifications and gene expression [276]. Further examples abound, providing valuable insights into the complexity of AS and its impact on gene regulation [244, 248, 277].

There are several splice variants of *METTL3*. Some variants are less catalytically active, leading to a reduction in the total amount of m6A in the cell and altering the ability of the cell to proliferate, migrate, and invade [278]. Not all AS variants have an effect on the m6A catalytic activity of METTL3. CRISPR/Cas9-based methods to remove *METTL3* can remove only about half of m6A because other *METTL3* splicing isoforms bypass the CRISPR/Cas9 mutation and produce functional methyltransferases [279]. A *METTL3* isoform that retains introns 8 and 9 has also been found in human tissues. The extended 3′UTR inhibits the transport of this isoform’s mRNA to the cytoplasm, thereby inhibiting protein expression [280]. Additionally, evidence suggests that *METTL14* undergoes AS, with the skipping or inclusion of exon 10 being regulated by the phosphorylation of SRSF5. Isoforms of *METTL14* that retain exon 10 exhibits higher catalytic activity [281].

A review of public databases revealed that approximately half of the epifactors possess at least one splice isoform, suggesting the presence of numerous yet unidentified isoforms [282]. These epifactors undergo AS to generate variant transcripts, which exhibit distinct functions in skeletal muscle atrophy, varying by tissue type and disease state. This raises several key questions for future research: What shifts in epifactors isoforms occur during skeletal muscle atrophy or regeneration? How do these isoforms compare in terms of their contributions to epigenetic modifications and physiological roles? What mechanisms of AS regulate the generation of these isoforms?

## Conclusion and perspectives

Understanding how atrophy genes or pathways are initiated following skeletal muscle atrophy is pivotal for developing strategies to delay this condition. Herein, we discussed the roles of epigenetic modifications of DNA, histones, and RNA, as well as their regulators, and provided insights into how epigenetic is an early signal that orchestrates the progression of skeletal muscle atrophy. Despite this, the functions of a limited number of epigenetic modifications and regulators have been systematically investigated in the context of skeletal muscle atrophy. A promising avenue for future research lies in elucidating the complex interplay between epigenetic mechanisms and alternative splicing and their collective impact on skeletal muscle atrophy. Moreover, the potential interactions between different forms of methylation (DNA, RNA, and histone) and between specific modifications such as 5mC and m6A in influencing skeletal muscle atrophy warrant further exploration [283, 284]. The interaction network of these epigenetic modifications in human skeletal muscle diseases and rodent models remains largely unclear.

Skeletal muscle exhibits considerable heterogeneity at various levels, including differences in the physiology and rate of atrophy between fast- and slow-twitch muscles, which may be under epigenetic control. For instance, the transcriptional states of type I, IIx, and IIb *MyHC* genes during *MyHC* conversion after muscle unloading show varying sensitivity to histone modifications [285]. Current insights into the epigenetic landscape of skeletal muscle are predominantly derived from bulk-omics data, potentially overlooking distinctions between fast and slow muscle myonuclei, muscle SCs, macrophages, and adipocytes. Recent attention to chromatin accessibility dynamics during skeletal muscle atrophy, at the resolution of single nuclei or fibers, represents a significant step forward. However, the dynamics of nuclear RNA and DNA methylation at such fine resolutions remain to be fully understood, offering a promising direction to unravel the complex regulation of skeletal muscle atrophy more profoundly [286].

With the advancement of epigenetics research, significant progress has been made in drug discovery and development [287]. The approval rate of epigenetic drugs for clinical use is increasing, with CRISPR–Cas-based epigenetic editing emerging as a groundbreaking approach in this field [288]. At present, most of these drugs function as inhibitors targeting epigenetic regulators, predominantly for cancer treatment, though a limited selection has been applied to combat skeletal muscle atrophy. Trichostatin A, derived from fungal metabolites and a potent histone deacetylase inhibitor, has demonstrated efficacy against skeletal muscle wasting and notable therapeutic outcomes in models of DMD, ALS, and SMA [192, 289]. Despite their potential, HDACI often encounter challenges in clinical development, primarily due to their wide-ranging effects and the lack of specificity in targeting downstream molecules, complicating the mitigation of pharmacological side effects. Thus, addressing this issue necessitates a comprehensive analysis of the molecular interaction networks of epigenetic drugs and a deeper understanding of their action mechanisms in specific physiological contexts.

## Data Availability

All data have been included in the manuscript.

## Abbreviations

DMD: Duchenne muscular dystrophy
ALS: Amyotrophic lateral sclerosis
SMA: Spinal muscular atrophy
MAFbx: Muscle atrophy F-box
MuRF1: Muscle RING Finger-1
TRAF6: TNF receptor associated factor 6
MyHC: Myosin heavy chain
SCs: Satellite cells
Pax7: Paired-box 7
5mC: 5-Methylcytosine
6 mA: N^6^-methyladenine
4mC: N^4^-methylcytosine
5hmC: 5-Hydroxymethylcytosine
DNMTs: DNA methyltransferases
SAM: *S*-adenosylmethionine
TET: Ten-eleven translocation
5fC: 5-Formylcytosine
5caC: 5-Carboxylcytosine
TDG: Thymine DNA glycosylase
PTMs: Post-translational modifications
HATs: Histone acetyltransferases
GNAT: Gcn5-associated *N*-acetyltransferase
TFIIIC: Transcription factor for RNA polymerase III
HDAC: Histone deacetylase
HMT: Histone methyltransferase
HDM: Histone demethylase
KMTs: Histone lysine methyltransferases
DOT1L: Disruptor of telomeric silencing 1-like
MLL: Mixed-lineage leukemia
EZH: Enhancer of zeste homolog
SMYD: SET and MYND domain-containing
PRDM: PR domain zinc finger protein
LSD1: Lysine-specific demethylase 1
FAD: Flavin adenine dinucleotide
JMJC: Jumonji C
mRNA: Messenger RNA
rRNA: Ribosomal RNA
tRNA: Transfer RNA
m6A: N^6^-methylation of adenosine
m1A: N^1^-methyladenine
m5C: 5-Methylcytosine
m3C: 3-Methylcytosine
m7G: 7-Methylguarine
m6Am: 2′-*O*-dimethyladenine nucleoside
CDS: Coding sequence
3′UTR: 3′-Untranslated region
5′UTR: 5′-Untranslated region
SRSF: Serine and arginine rich splicing factor
METTL: Methyltransferase-like
WTAP: Wilms tumor 1 associated protein
VIRMA: Vir like m6A methyltransferase associated
ALKBH5: Alpha-ketoglutarate-dependent dioxygenase alkB homolog 5
FTO: Fat mass and obesity-associated protein
NMJ: Neuromuscular junction
HNRNP: Heterogeneous nuclear ribonucleoprotein
TBCD: Tubulin folding cofactor D
TTX: Tetrodotoxin
ICU: Intensive care unit
KO: Knockout
Gdf5: Growth differentiation factor 5
CDKIs: Cyclin-dependent kinase inhibitors
TWEAK: TNF-like weak inducer of apoptosis
Fn14: Fibroblast growth factor-inducible 14
DM: Diabetes mellitus
STZ: Streptozotocin
EDL: Extensor digitorum longus
MMP: Matrix metalloproteinases
COPD: Chronic obstructive pulmonary disease
AMPK: Adenosine 5′-monophosphate (AMP)-activated protein kinase
SCF: SKP1-cullin1-F-box
CARM1: Coactivator-associated arginine methyltransferase 1
FOXO3: Forkhead box O3
HIF-1α: Hypoxia-inducible transcription factor-1α
KDM: Lysine-specific demethylase
mTOR: Mammalian target of rapamycin
NF-κB: Nuclear factor kappa-B
TGF-β: Transforming growth factor β
CBP: CREB-binding protein
KLF5: Krüppel-like factor 5
ceRNA: Competitive endogenous RNA
TSA: Trichostatin A
R-2HG: *R*-2-hydroxyglutarate
Daa: Deazadenosine
RBP: RNA-binding protein
HCEs: Highly conserved exons
AS: Alternative splicing
HP1: Heterochromatin protein 1
MRG15: MORF-related gene 15
CTCF: CCCTC-binding factor

## References

[1] Yin L, Li N, Jia W, Wang N, Liang M, Yang X, Du G (2021). Skeletal muscle atrophy: from mechanisms to treatments. Pharmacol Res.

[2] Sartori R, Romanello V, Sandri M (2021). Mechanisms of muscle atrophy and hypertrophy: implications in health and disease. Nat Commun.

[3] Furrer R, Handschin C (2019). Muscle wasting diseases: novel targets and treatments. Annu Rev Pharmacol Toxicol.

[4] Davegardh C, Sall J, Benrick A, Broholm C, Volkov P, Perfilyev A, Henriksen TI, Wu Y, Hjort L, Brons C (2021). VPS39-deficiency observed in type 2 diabetes impairs muscle stem cell differentiation via altered autophagy and epigenetics. Nat Commun.

[5] Bilgic SN, Domaniku A, Toledo B, Agca S, Weber BZC, Arabaci DH, Ozornek Z, Lause P, Thissen JP, Loumaye A (2023). EDA2R-NIK signalling promotes muscle atrophy linked to cancer cachexia. Nature.

[6] Mercuri E, Sumner CJ, Muntoni F, Darras BT, Finkel RS (2022). Spinal muscular atrophy. Nat Rev Dis Primers.

[7] Wilkinson DJ, Piasecki M, Atherton PJ (2018). The age-related loss of skeletal muscle mass and function: measurement and physiology of muscle fibre atrophy and muscle fibre loss in humans. Ageing Res Rev.

[8] Jin Y, Song Y, Lin J, Liu T, Li G, Lai B, Gu Y, Chen G, Xing L (2023). Role of inflammation in neurological damage and regeneration following spinal cord injury and its therapeutic implications. Burns Trauma.

[9] Zhang J, Gao Y, Yan J (2024). Roles of myokines and muscle-derived extracellular vesicles in musculoskeletal deterioration under disuse conditions. Metabolites.

[10] Bodine SC, Latres E, Baumhueter S, Lai VK, Nunez L, Clarke BA, Poueymirou WT, Panaro FJ, Na E, Dharmarajan K (2001). Identification of ubiquitin ligases required for skeletal muscle atrophy. Science.

[11] Gomes MD, Lecker SH, Jagoe RT, Navon A, Goldberg AL (2001). Atrogin-1, a muscle-specific F-box protein highly expressed during muscle atrophy. Proc Natl Acad Sci USA.

[12] Qiu J, Zhu J, Zhang R, Liang W, Ma W, Zhang Q, Huang Z, Ding F, Sun H (2019). miR-125b-5p targeting TRAF6 relieves skeletal muscle atrophy induced by fasting or denervation. Ann Transl Med.

[13] Qaisar R, Bhaskaran S, Van Remmen H (2016). Muscle fiber type diversification during exercise and regeneration. Free Radic Biol Med.

[14] Wang Y, Pessin JE (2013). Mechanisms for fiber-type specificity of skeletal muscle atrophy. Curr Opin Clin Nutr Metab Care.

[15] Ciciliot S, Rossi AC, Dyar KA, Blaauw B, Schiaffino S (2013). Muscle type and fiber type specificity in muscle wasting. Int J Biochem Cell Biol.

[16] Talbot J, Maves L (2016). Skeletal muscle fiber type: using insights from muscle developmental biology to dissect targets for susceptibility and resistance to muscle disease. Wiley Interdiscip Rev Dev Biol.

[17] Kahn RE, Dayanidhi S, Lacham-Kaplan O, Hawley JA (2023). Molecular clocks, satellite cells, and skeletal muscle regeneration. Am J Physiol Cell Physiol.

[18] Chervu A, Moore WS, Chvapil M, Henderson T (1991). Efficacy and duration of antistaphylococcal activity comparing three antibiotics bonded to Dacron vascular grafts with a collagen release system. J Vasc Surg.

[19] Choo CS, Chen Y, McHoney M (2022). Delayed versus early repair of inguinal hernia in preterm infants: a systematic review and meta-analysis. J Pediatr Surg.

[20] Hernandez-Hernandez JM, Garcia-Gonzalez EG, Brun CE, Rudnicki MA (2017). The myogenic regulatory factors, determinants of muscle development, cell identity and regeneration. Semin Cell Dev Biol.

[21] Goldberg AD, Allis CD, Bernstein E (2007). Epigenetics: a landscape takes shape. Cell.

[22] Fitz-James MH, Cavalli G (2022). Molecular mechanisms of transgenerational epigenetic inheritance. Nat Rev Genet.

[23] Bianconi V, Mozzetta C (2022). Epigenetic control of muscle stem cells: time for a new dimension. Trends Genet.

[24] Dilworth FJ, Blais A (2011). Epigenetic regulation of satellite cell activation during muscle regeneration. Stem Cell Res Ther.

[25] Sahinyan K, Blackburn DM, Simon MM, Lazure F, Kwan T, Bourque G, Soleimani VD (2022). Application of ATAC-Seq for genome-wide analysis of the chromatin state at single myofiber resolution. Elife.

[26] Allis CD, Jenuwein T (2016). The molecular hallmarks of epigenetic control. Nat Rev Genet.

[27] Zhao LY, Song J, Liu Y, Song CX, Yi C (2020). Mapping the epigenetic modifications of DNA and RNA. Protein Cell.

[28] Kottakis F, Nicolay BN, Roumane A, Karnik R, Gu H, Nagle JM, Boukhali M, Hayward MC, Li YY, Chen T (2016). LKB1 loss links serine metabolism to DNA methylation and tumorigenesis. Nature.

[29] Moore LD, Le T, Fan G (2013). DNA methylation and its basic function. Neuropsychopharmacology.

[30] Raddatz G, Guzzardo PM, Olova N, Fantappie MR, Rampp M, Schaefer M, Reik W, Hannon GJ, Lyko F (2013). Dnmt2-dependent methylomes lack defined DNA methylation patterns. Proc Natl Acad Sci USA.

[31] Mattei AL, Bailly N, Meissner A (2022). DNA methylation: a historical perspective. Trends Genet.

[32] Greenberg MVC, Bourc'his D (2019). The diverse roles of DNA methylation in mammalian development and disease. Nat Rev Mol Cell Biol.

[33] Tajima S, Suetake I, Takeshita K, Nakagawa A, Kimura H (2016). Domain structure of the Dnmt1, Dnmt3a, and Dnmt3b DNA methyltransferases. Adv Exp Med Biol.

[34] Barau J, Teissandier A, Zamudio N, Roy S, Nalesso V, Herault Y, Guillou F, Bourc'his D (2016). The DNA methyltransferase DNMT3C protects male germ cells from transposon activity. Science.

[35] Wu X, Zhang Y (2017). TET-mediated active DNA demethylation: mechanism, function and beyond. Nat Rev Genet.

[36] Illingworth R, Kerr A, Desousa D, Jorgensen H, Ellis P, Stalker J, Jackson D, Clee C, Plumb R, Rogers J (2008). A novel CpG island set identifies tissue-specific methylation at developmental gene loci. PLoS Biol.

[37] Sorensen AL, Jacobsen BM, Reiner AH, Andersen IS, Collas P (2010). Promoter DNA methylation patterns of differentiated cells are largely programmed at the progenitor stage. Mol Biol Cell.

[38] Maehara H, Kokaji T, Hatano A, Suzuki Y, Matsumoto M, Nakayama KI, Egami R, Tsuchiya T, Ozaki H, Morita K (2023). DNA hypomethylation characterizes genes encoding tissue-dominant functional proteins in liver and skeletal muscle. Sci Rep.

[39] Calvanese V, Fernandez AF, Urdinguio RG, Suarez-Alvarez B, Mangas C, Perez-Garcia V, Bueno C, Montes R, Ramos-Mejia V, Martinez-Camblor P (2012). A promoter DNA demethylation landscape of human hematopoietic differentiation. Nucleic Acids Res.

[40] Tsumagari K, Baribault C, Terragni J, Varley KE, Gertz J, Pradhan S, Badoo M, Crain CM, Song L, Crawford GE (2013). Early de novo DNA methylation and prolonged demethylation in the muscle lineage. Epigenetics.

[41] Zykovich A, Hubbard A, Flynn JM, Tarnopolsky M, Fraga MF, Kerksick C, Ogborn D, MacNeil L, Mooney SD, Melov S (2014). Genome-wide DNA methylation changes with age in disease-free human skeletal muscle. Aging Cell.

[42] Turner DC, Gorski PP, Maasar MF, Seaborne RA, Baumert P, Brown AD, Kitchen MO, Erskine RM, Dos-Remedios I, Voisin S (2020). DNA methylation across the genome in aged human skeletal muscle tissue and muscle-derived cells: the role of HOX genes and physical activity. Sci Rep.

[43] Gensous N, Bacalini MG, Franceschi C, Meskers CGM, Maier AB, Garagnani P (2019). Age-related DNA methylation changes: potential impact on skeletal muscle aging in humans. Front Physiol.

[44] Carter HN, Pauly M, Tryon LD, Hood DA (1985). Effect of contractile activity on PGC-1alpha transcription in young and aged skeletal muscle. J Appl Physiol.

[45] Voisin S, Jacques M, Landen S, Harvey NR, Haupt LM, Griffiths LR, Gancheva S, Ouni M, Jahnert M, Ashton KJ (2021). Meta-analysis of genome-wide DNA methylation and integrative omics of age in human skeletal muscle. J Cachexia Sarcopenia Muscle.

[46] Murach KA, Dimet-Wiley AL, Wen Y, Brightwell CR, Latham CM, Dungan CM, Fry CS, Watowich SJ (2022). Late-life exercise mitigates skeletal muscle epigenetic aging. Aging Cell.

[47] Brown WM (2015). Exercise-associated DNA methylation change in skeletal muscle and the importance of imprinted genes: a bioinformatics meta-analysis. Br J Sports Med.

[48] Fisher AG, Seaborne RA, Hughes TM, Gutteridge A, Stewart C, Coulson JM, Sharples AP, Jarvis JC (2017). Transcriptomic and epigenetic regulation of disuse atrophy and the return to activity in skeletal muscle. FASEB J.

[49] Van Dyck L, Guiza F, Derese I, Pauwels L, Casaer MP, Hermans G, Wouters PJ, Van den Berghe G, Vanhorebeek I (2022). DNA methylation alterations in muscle of critically ill patients. J Cachexia Sarcopenia Muscle.

[50] Hatazawa Y, Ono Y, Hirose Y, Kanai S, Fujii NL, Machida S, Nishino I, Shimizu T, Okano M, Kamei Y (2018). Reduced Dnmt3a increases Gdf5 expression with suppressed satellite cell differentiation and impaired skeletal muscle regeneration. FASEB J.

[51] Small L, Ingerslev LR, Manitta E, Laker RC, Hansen AN, Deeney B, Carrie A, Couvert P, Barres R (2021). Ablation of DNA-methyltransferase 3A in skeletal muscle does not affect energy metabolism or exercise capacity. PLoS Genet.

[52] Naito M, Mori M, Inagawa M, Miyata K, Hashimoto N, Tanaka S, Asahara H (2016). Dnmt3a regulates proliferation of muscle satellite cells via p57Kip2. PLoS Genet.

[53] Mittal A, Bhatnagar S, Kumar A, Lach-Trifilieff E, Wauters S, Li H, Makonchuk DY, Glass DJ, Kumar A (2010). The TWEAK-Fn14 system is a critical regulator of denervation-induced skeletal muscle atrophy in mice. J Cell Biol.

[54] Tajrishi MM, Shin J, Hetman M, Kumar A (2014). DNA methyltransferase 3a and mitogen-activated protein kinase signaling regulate the expression of fibroblast growth factor-inducible 14 (Fn14) during denervation-induced skeletal muscle atrophy. J Biol Chem.

[55] Wang M, Wu X, Gan L, Teng Z, Zhang H, Zhang Y (2020). Overexpression of Dnmt3a ameliorates diabetic muscle atrophy by modulating the Pten/Akt pathway. Exp Physiol.

[56] Martin LJ, Adams DA, Niedzwiecki MV, Wong M (2022). Aberrant DNA and RNA methylation occur in spinal cord and skeletal muscle of human SOD1 mouse models of ALS and in human ALS: targeting DNA METHYLATION IS THERApeutic. Cells.

[57] Kondo N, Tohnai G, Sahashi K, Iida M, Kataoka M, Nakatsuji H, Tsutsumi Y, Hashizume A, Adachi H, Koike H (2019). DNA methylation inhibitor attenuates polyglutamine-induced neurodegeneration by regulating Hes5. EMBO Mol Med.

[58] Wong M, Gertz B, Chestnut BA, Martin LJ (2013). Mitochondrial DNMT3A and DNA methylation in skeletal muscle and CNS of transgenic mouse models of ALS. Front Cell Neurosci.

[59] Liu R, Kim KY, Jung YW, Park IH (2016). Dnmt1 regulates the myogenic lineage specification of muscle stem cells. Sci Rep.

[60] Gu TP, Guo F, Yang H, Wu HP, Xu GF, Liu W, Xie ZG, Shi L, He X, Jin SG (2011). The role of Tet3 DNA dioxygenase in epigenetic reprogramming by oocytes. Nature.

[61] Carrio E, Suelves M (2015). DNA methylation dynamics in muscle development and disease. Front Aging Neurosci.

[62] Dawlaty MM, Breiling A, Le T, Barrasa MI, Raddatz G, Gao Q, Powell BE, Cheng AW, Faull KF, Lyko F (2014). Loss of Tet enzymes compromises proper differentiation of embryonic stem cells. Dev Cell.

[63] Zhang H, Wang S, Zhou Q, Liao Y, Luo W, Peng Z, Ren R, Wang H (2022). Disturbance of calcium homeostasis and myogenesis caused by TET2 deletion in muscle stem cells. Cell Death Discov.

[64] Wang H, Huang Y, Yu M, Yu Y, Li S, Wang H, Sun H, Li B, Xu G, Hu P (2021). Muscle regeneration controlled by a designated DNA dioxygenase. Cell Death Dis.

[65] Zhang M, Chen M, Li Y, Rao M, Wang D, Wang Z, Zhang L, Yin P, Tang P (2023). Delayed denervation-induced muscle atrophy in Opg knockout mice. Front Physiol.

[66] Meng X, Tian C, Xie C, Zhang H, Wang H, Zhang M, Lu Z, Li D, Chen L, Gao T (2023). Punicalagin protects against impaired skeletal muscle function in high-fat-diet-induced obese mice by regulating TET2. Food Funct.

[67] Zhong X, Wang QQ, Li JW, Zhang YM, An XR, Hou J (2017). Ten-Eleven translocation-2 (Tet2) is involved in myogenic differentiation of skeletal myoblast cells in vitro. Sci Rep.

[68] Zhang T, Guan X, Choi UL, Dong Q, Lam MMT, Zeng J, Xiong J, Wang X, Poon TCW, Zhang H (2019). Phosphorylation of TET2 by AMPK is indispensable in myogenic differentiation. Epigenetics Chromatin.

[69] Nitsch S, Zorro Shahidian L, Schneider R (2021). Histone acylations and chromatin dynamics: concepts, challenges, and links to metabolism. EMBO Rep.

[70] Hagihara H, Shoji H, Otabi H, Toyoda A, Katoh K, Namihira M, Miyakawa T (2021). Protein lactylation induced by neural excitation. Cell Rep.

[71] Lepack AE, Werner CT, Stewart AF, Fulton SL, Zhong P, Farrelly LA, Smith ACW, Ramakrishnan A, Lyu Y, Bastle RM (2020). Dopaminylation of histone H3 in ventral tegmental area regulates cocaine seeking. Science.

[72] Farrelly LA, Thompson RE, Zhao S, Lepack AE, Lyu Y, Bhanu NV, Zhang B, Loh YE, Ramakrishnan A, Vadodaria KC (2019). Histone serotonylation is a permissive modification that enhances TFIID binding to H3K4me3. Nature.

[73] Bannister AJ, Kouzarides T (2011). Regulation of chromatin by histone modifications. Cell Res.

[74] Audia JE, Campbell RM (2016). Histone modifications and cancer. Cold Spring Harb Perspect Biol.

[75] Park J, Lee K, Kim K, Yi SJ (2022). The role of histone modifications: from neurodevelopment to neurodiseases. Signal Transduct Target Ther.

[76] Marmorstein R, Zhou MM (2014). Writers and readers of histone acetylation: structure, mechanism, and inhibition. Cold Spring Harb Perspect Biol.

[77] Yang XJ, Seto E (2007). HATs and HDACs: from structure, function and regulation to novel strategies for therapy and prevention. Oncogene.

[78] Wapenaar H, Dekker FJ (2016). Histone acetyltransferases: challenges in targeting bi-substrate enzymes. Clin Epigenetics.

[79] Xu J, Li C, Kang X (2023). The epigenetic regulatory effect of histone acetylation and deacetylation on skeletal muscle metabolism-a review. Front Physiol.

[80] Vezzoli M, de Llobet Cucalon LI, Di Vona C, Morselli M, Montanini B, de la Luna S, Teichmann M, Dieci G, Ferrari R (2023). TFIIIC as a potential epigenetic modulator of histone acetylation in human stem cells. Int J Mol Sci.

[81] Doi M, Hirayama J, Sassone-Corsi P (2006). Circadian regulator CLOCK is a histone acetyltransferase. Cell.

[82] Seto E, Yoshida M (2014). Erasers of histone acetylation: the histone deacetylase enzymes. Cold Spring Harb Perspect Biol.

[83] Greer EL, Shi Y (2012). Histone methylation: a dynamic mark in health, disease and inheritance. Nat Rev Genet.

[84] Tan M, Luo H, Lee S, Jin F, Yang JS, Montellier E, Buchou T, Cheng Z, Rousseaux S, Rajagopal N (2011). Identification of 67 histone marks and histone lysine crotonylation as a new type of histone modification. Cell.

[85] Martin C, Zhang Y (2005). The diverse functions of histone lysine methylation. Nat Rev Mol Cell Biol.

[86] Kouzarides T (2007). Chromatin modifications and their function. Cell.

[87] Trievel RC, Beach BM, Dirk LM, Houtz RL, Hurley JH (2002). Structure and catalytic mechanism of a SET domain protein methyltransferase. Cell.

[88] Allis CD, Berger SL, Cote J, Dent S, Jenuwien T, Kouzarides T, Pillus L, Reinberg D, Shi Y, Shiekhattar R (2007). New nomenclature for chromatin-modifying enzymes. Cell.

[89] Mosammaparast N, Shi Y (2010). Reversal of histone methylation: biochemical and molecular mechanisms of histone demethylases. Annu Rev Biochem.

[90] Yoshihara T, Machida S, Tsuzuki T, Kakigi R, Chang SW, Sugiura T, Naito H (2019). Age-related changes in histone modification in rat gastrocnemius muscle. Exp Gerontol.

[91] Ryder DJ, Judge SM, Beharry AW, Farnsworth CL, Silva JC, Judge AR (2015). Identification of the acetylation and ubiquitin-modified proteome during the progression of skeletal muscle atrophy. PLoS ONE.

[92] Kawano F, Nimura K, Ishino S, Nakai N, Nakata K, Ohira Y (2015). Differences in histone modifications between slow- and fast-twitch muscle of adult rats and following overload, denervation, or valproic acid administration. J Appl Physiol (1985).

[93] Ramachandran K, Senagolage MD, Sommars MA, Futtner CR, Omura Y, Allred AL, Barish GD (2019). Dynamic enhancers control skeletal muscle identity and reprogramming. PLoS Biol.

[94] Ohsawa I, Kawano F (2021). Chronic exercise training activates histone turnover in mouse skeletal muscle fibers. FASEB J.

[95] Williams K, Carrasquilla GD, Ingerslev LR, Hochreuter MY, Hansson S, Pillon NJ, Donkin I, Versteyhe S, Zierath JR, Kilpelainen TO (2021). Epigenetic rewiring of skeletal muscle enhancers after exercise training supports a role in whole-body function and human health. Mol Metab.

[96] Zhong Q, Zheng K, Li W, An K, Liu Y, Xiao X, Hai S, Dong B, Li S, An Z (2023). Post-translational regulation of muscle growth, muscle aging and sarcopenia. J Cachexia Sarcopenia Muscle.

[97] Masuzawa R, Konno R, Ohsawa I, Watanabe A, Kawano F (2018). Muscle type-specific RNA polymerase II recruitment during PGC-1alpha gene transcription after acute exercise in adult rats. J Appl Physiol.

[98] Sandri M, Lin J, Handschin C, Yang W, Arany ZP, Lecker SH, Goldberg AL, Spiegelman BM (2006). PGC-1alpha protects skeletal muscle from atrophy by suppressing FoxO3 action and atrophy-specific gene transcription. Proc Natl Acad Sci USA.

[99] Cramer AAW, Prasad V, Eftestol E, Song T, Hansson KA, Dugdale HF, Sadayappan S, Ochala J, Gundersen K, Millay DP (2020). Nuclear numbers in syncytial muscle fibers promote size but limit the development of larger myonuclear domains. Nat Commun.

[100] Sahinyan K, Blackburn DM, Soleimani VD (2022). ATAC-Seq of a Single Myofiber from Mus musculus. Bio Protoc.

[101] Blackburn DM, Lazure F, Corchado AH, Perkins TJ, Najafabadi HS, Soleimani VD (2019). High-resolution genome-wide expression analysis of single myofibers using SMART-Seq. J Biol Chem.

[102] Mal AK (2006). Histone methyltransferase Suv39h1 represses MyoD-stimulated myogenic differentiation. EMBO J.

[103] Chatterjee B, Wolff DW, Jothi M, Mal M, Mal AK (2016). p38alpha MAPK disables KMT1A-mediated repression of myogenic differentiation program. Skelet Muscle.

[104] Collins R, Cheng X (2010). A case study in cross-talk: the histone lysine methyltransferases G9a and GLP. Nucleic Acids Res.

[105] Tachibana M, Sugimoto K, Nozaki M, Ueda J, Ohta T, Ohki M, Fukuda M, Takeda N, Niida H, Kato H (2002). G9a histone methyltransferase plays a dominant role in euchromatic histone H3 lysine 9 methylation and is essential for early embryogenesis. Genes Dev.

[106] Tachibana M, Ueda J, Fukuda M, Takeda N, Ohta T, Iwanari H, Sakihama T, Kodama T, Hamakubo T, Shinkai Y (2005). Histone methyltransferases G9a and GLP form heteromeric complexes and are both crucial for methylation of euchromatin at H3–K9. Genes Dev.

[107] Balemans MC, Ansar M, Oudakker AR, van Caam AP, Bakker B, Vitters EL, van der Kraan PM, de Bruijn DR, Janssen SM, Kuipers AJ (2014). Reduced Euchromatin histone methyltransferase 1 causes developmental delay, hypotonia, and cranial abnormalities associated with increased bone gene expression in Kleefstra syndrome mice. Dev Biol.

[108] Biferali B, Bianconi V, Perez DF, Kronawitter SP, Marullo F, Maggio R, Santini T, Polverino F, Biagioni S, Summa V (2021). Prdm16-mediated H3K9 methylation controls fibro-adipogenic progenitors identity during skeletal muscle repair. Sci Adv.

[109] Zhang RH, Judson RN, Liu DY, Kast J, Rossi FM (2016). The lysine methyltransferase Ehmt2/G9a is dispensable for skeletal muscle development and regeneration. Skelet Muscle.

[110] Palacios D, Puri PL (2006). The epigenetic network regulating muscle development and regeneration. J Cell Physiol.

[111] Acharyya S, Sharma SM, Cheng AS, Ladner KJ, He W, Kline W, Wang H, Ostrowski MC, Huang TH, Guttridge DC (2010). TNF inhibits Notch-1 in skeletal muscle cells by Ezh2 and DNA methylation mediated repression: implications in duchenne muscular dystrophy. PLoS ONE.

[112] Acharyya S, Villalta SA, Bakkar N, Bupha-Intr T, Janssen PM, Carathers M, Li ZW, Beg AA, Ghosh S, Sahenk Z (2007). Interplay of IKK/NF-kappaB signaling in macrophages and myofibers promotes muscle degeneration in Duchenne muscular dystrophy. J Clin Invest.

[113] Consalvi S, Brancaccio A, Dall'Agnese A, Puri PL, Palacios D (2017). Praja1 E3 ubiquitin ligase promotes skeletal myogenesis through degradation of EZH2 upon p38alpha activation. Nat Commun.

[114] Feng X, Wang AH, Juan AH, Ko KD, Jiang K, Riparini G, Ciuffoli V, Kaba A, Lopez C, Naz F (2023). Polycomb Ezh1 maintains murine muscle stem cell quiescence through non-canonical regulation of Notch signaling. Dev Cell.

[115] Lu X, Liang B, Li S, Chen Z, Chang W (2020). Modulation of HOXA9 after skeletal muscle denervation and reinnervation. Am J Physiol Cell Physiol.

[116] Addicks GC, Brun CE, Sincennes MC, Saber J, Porter CJ, Francis Stewart A, Ernst P, Rudnicki MA (2019). MLL1 is required for PAX7 expression and satellite cell self-renewal in mice. Nat Commun.

[117] Sebastian S, Sreenivas P, Sambasivan R, Cheedipudi S, Kandalla P, Pavlath GK, Dhawan J (2009). MLL5, a trithorax homolog, indirectly regulates H3K4 methylation, represses cyclin A2 expression, and promotes myogenic differentiation. Proc Natl Acad Sci U S A.

[118] de Esteves Lima J, Bou Akar R, Machado L, Li Y, Drayton-Libotte B, Dilworth FJ, Relaix F (2021). HIRA stabilizes skeletal muscle lineage identity. Nat Commun.

[119] Cai S, Zhu Q, Guo C, Yuan R, Zhang X, Nie Y, Chen L, Fang Y, Chen K, Zhang J (2020). MLL1 promotes myogenesis by epigenetically regulating Myf5. Cell Prolif.

[120] Vicente-Garcia C, Hernandez-Camacho JD, Carvajal JJ (2022). Regulation of myogenic gene expression. Exp Cell Res.

[121] Rampalli S, Li L, Mak E, Ge K, Brand M, Tapscott SJ, Dilworth FJ (2007). p38 MAPK signaling regulates recruitment of Ash2L-containing methyltransferase complexes to specific genes during differentiation. Nat Struct Mol Biol.

[122] Liu L, Ding C, Fu T, Feng Z, Lee JE, Xiao L, Xu Z, Yin Y, Guo Q, Sun Z (2020). Histone methyltransferase MLL4 controls myofiber identity and muscle performance through MEF2 interaction. J Clin Invest.

[123] Gao J, Li J, Li BJ, Yagil E, Zhang J, Du SJ (2014). Expression and functional characterization of Smyd1a in myofibril organization of skeletal muscles. PLoS ONE.

[124] Proserpio V, Fittipaldi R, Ryall JG, Sartorelli V, Caretti G (2013). The methyltransferase SMYD3 mediates the recruitment of transcriptional cofactors at the myostatin and c-Met genes and regulates skeletal muscle atrophy. Genes Dev.

[125] Stewart MD, Lopez S, Nagandla H, Soibam B, Benham A, Nguyen J, Valenzuela N, Wu HJ, Burns AR, Rasmussen TL (2016). Mouse myofibers lacking the SMYD1 methyltransferase are susceptible to atrophy, internalization of nuclei and myofibrillar disarray. Dis Model Mech.

[126] Munkanatta Godage DNP, VanHecke GC, Samarasinghe KTG, Feng HZ, Hiske M, Holcomb J, Yang Z, Jin JP, Chung CS, Ahn YH (2018). SMYD2 glutathionylation contributes to degradation of sarcomeric proteins. Nat Commun.

[127] Nguyen AT, Xiao B, Neppl RL, Kallin EM, Li J, Chen T, Wang DZ, Xiao X, Zhang Y (2011). DOT1L regulates dystrophin expression and is critical for cardiac function. Genes Dev.

[128] Lakhdar R, Drost EM, MacNee W, Bastos R, Rabinovich RA (2017). 2D-DIGE proteomic analysis of vastus lateralis from COPD patients with low and normal fat free mass index and healthy controls. Respir Res.

[129] vanLieshout TL, Ljubicic V (2019). The emergence of protein arginine methyltransferases in skeletal muscle and metabolic disease. Am J Physiol Endocrinol Metab.

[130] Blanc RS, Vogel G, Li X, Yu Z, Li S, Richard S (2017). Arginine methylation by PRMT1 regulates muscle stem cell fate. Mol Cell Biol.

[131] Dhar S, Vemulapalli V, Patananan AN, Huang GL, Di Lorenzo A, Richard S, Comb MJ, Guo A, Clarke SG, Bedford MT (2013). Loss of the major Type I arginine methyltransferase PRMT1 causes substrate scavenging by other PRMTs. Sci Rep.

[132] Pawlak MR, Scherer CA, Chen J, Roshon MJ, Ruley HE (2000). Arginine N-methyltransferase 1 is required for early postimplantation mouse development, but cells deficient in the enzyme are viable. Mol Cell Biol.

[133] Stouth DW, vanLieshout TL, Shen NY, Ljubicic V (2017). Regulation of skeletal muscle plasticity by protein arginine methyltransferases and their potential roles in neuromuscular disorders. Front Physiol.

[134] Yu Z, Chen T, Hebert J, Li E, Richard S (2009). A mouse PRMT1 null allele defines an essential role for arginine methylation in genome maintenance and cell proliferation. Mol Cell Biol.

[135] Choi S, Jeong HJ, Kim H, Choi D, Cho SC, Seong JK, Koo SH, Kang JS (2019). Skeletal muscle-specific Prmt1 deletion causes muscle atrophy via deregulation of the PRMT6-FOXO3 axis. Autophagy.

[136] Liu Y, Li J, Shang Y, Guo Y, Li Z (2019). CARM1 contributes to skeletal muscle wasting by mediating FoxO3 activity and promoting myofiber autophagy. Exp Cell Res.

[137] Blanc RS, Vogel G, Chen T, Crist C, Richard S (2016). PRMT7 preserves satellite cell regenerative capacity. Cell Rep.

[138] Matsui F, Watanabe E, Oohira A (1994). Immunological identification of two proteoglycan fragments derived from neurocan, a brain-specific chondroitin sulfate proteoglycan. Neurochem Int.

[139] Zhang T, Gunther S, Looso M, Kunne C, Kruger M, Kim J, Zhou Y, Braun T (2015). Prmt5 is a regulator of muscle stem cell expansion in adult mice. Nat Commun.

[140] Jeong HJ, Lee SJ, Lee HJ, Kim HB, Anh Vuong T, Cho H, Bae GU, Kang JS (2020). Prmt7 promotes myoblast differentiation via methylation of p38MAPK on arginine residue 70. Cell Death Differ.

[141] Stouth DW, Manta A, Ljubicic V (2018). Protein arginine methyltransferase expression, localization, and activity during disuse-induced skeletal muscle plasticity. Am J Physiol Cell Physiol.

[142] Jeong HJ, Lee HJ, Vuong TA, Choi KS, Choi D, Koo SH, Cho SC, Cho H, Kang JS (2016). Prmt7 deficiency causes reduced skeletal muscle oxidative metabolism and age-related obesity. Diabetes.

[143] So HK, Kim S, Kang JS, Lee SJ (2021). Role of protein arginine methyltransferases and inflammation in muscle pathophysiology. Front Physiol.

[144] Shin HJ, Kim H, Oh S, Lee JG, Kee M, Ko HJ, Kweon MN, Won KJ, Baek SH (2016). AMPK-SKP2-CARM1 signalling cascade in transcriptional regulation of autophagy. Nature.

[145] Cicciarello D, Schaeffer L, Scionti I (2022). Epigenetic control of muscle stem cells: focus on histone lysine demethylases. Front Cell Dev Biol.

[146] Sakaguchi M, Cai W, Wang CH, Cederquist CT, Damasio M, Homan EP, Batista T, Ramirez AK, Gupta MK, Steger M (2019). FoxK1 and FoxK2 in insulin regulation of cellular and mitochondrial metabolism. Nat Commun.

[147] Bowman CJ, Ayer DE, Dynlacht BD (2014). Foxk proteins repress the initiation of starvation-induced atrophy and autophagy programs. Nat Cell Biol.

[148] Araki H, Hino S, Anan K, Kuribayashi K, Etoh K, Seko D, Takase R, Kohrogi K, Hino Y, Ono Y (2023). LSD1 defines the fiber type-selective responsiveness to environmental stress in skeletal muscle. Elife.

[149] Schakman O, Kalista S, Barbe C, Loumaye A, Thissen JP (2013). Glucocorticoid-induced skeletal muscle atrophy. Int J Biochem Cell Biol.

[150] Salminen A, Kaarniranta K, Kauppinen A (2016). Hypoxia-inducible histone lysine demethylases: impact on the aging process and age-related diseases. Aging Dis.

[151] Salminen A, Kaarniranta K, Hiltunen M, Kauppinen A (2014). Histone demethylase Jumonji D3 (JMJD3/KDM6B) at the nexus of epigenetic regulation of inflammation and the aging process. J Mol Med (Berl).

[152] Liu X, Greer C, Secombe J (2014). KDM5 interacts with Foxo to modulate cellular levels of oxidative stress. PLoS Genet.

[153] Wu Z, Lu M, Liu D, Shi Y, Ren J, Wang S, Jing Y, Zhang S, Zhao Q, Li H (2023). m(6)A epitranscriptomic regulation of tissue homeostasis during primate aging. Nat Aging.

[154] Kudou K, Komatsu T, Nogami J, Maehara K, Harada A, Saeki H, Oki E, Maehara Y, Ohkawa Y (2017). The requirement of Mettl3-promoted MyoD mRNA maintenance in proliferative myoblasts for skeletal muscle differentiation. Open Biol.

[155] Deng K, Liu Z, Li X, Ren C, Fan Y, Guo J, Li P, Deng M, Xue G, Yu X (2024). Ythdf2-mediated STK11 mRNA decay supports myogenesis by inhibiting the AMPK/mTOR pathway. Int J Biol Macromol.

[156] Zhao T, Zhao R, Yi X, Cai R, Pang W (2022). METTL3 promotes proliferation and myogenic differentiation through m(6)A RNA methylation/YTHDF1/2 signaling axis in myoblasts. Life Sci.

[157] Liu Y, Zhou T, Wang Q, Fu R, Zhang Z, Chen N, Li Z, Gao G, Peng S, Yang D (2022). m(6) A demethylase ALKBH5 drives denervation-induced muscle atrophy by targeting HDAC4 to activate FoxO3 signalling. J Cachexia Sarcopenia Muscle.

[158] Ye M, Chen J, Lu F, Zhao M, Wu S, Hu C, Yu P, Kan J, Bai J, Tian Y (2023). Down-regulated FTO and ALKBH5 co-operatively activates FOXO signaling through m6A methylation modification in HK2 mRNA mediated by IGF2BP2 to enhance glycolysis in colorectal cancer. Cell Biosci.

[159] Wang X, Huang N, Yang M, Wei D, Tai H, Han X, Gong H, Zhou J, Qin J, Wei X (2017). FTO is required for myogenesis by positively regulating mTOR-PGC-1alpha pathway-mediated mitochondria biogenesis. Cell Death Dis.

[160] Deng K, Liu Z, Li X, Zhang Z, Fan Y, Huang Q, Zhang Y, Wang F (2023). Targeted demethylation of the TGFbeta1 mRNA promotes myoblast proliferation via activating the SMAD2 signaling pathway. Cells.

[161] Vanlieshout TL, Stouth DW, Tajik T, Ljubicic V (2018). Exercise-induced protein arginine methyltransferase expression in skeletal muscle. Med Sci Sports Exerc.

[162] Sin TK, Zhu JZ, Zhang G, Li YP (2019). p300 mediates muscle wasting in Lewis lung carcinoma. Cancer Res.

[163] Zhang G, Jin B, Li YP (2011). C/EBPbeta mediates tumour-induced ubiquitin ligase atrogin1/MAFbx upregulation and muscle wasting. EMBO J.

[164] Senf SM, Sandesara PB, Reed SA, Judge AR (2011). p300 Acetyltransferase activity differentially regulates the localization and activity of the FOXO homologues in skeletal muscle. Am J Physiol Cell Physiol.

[165] Beharry AW, Sandesara PB, Roberts BM, Ferreira LF, Senf SM, Judge AR (2014). HDAC1 activates FoxO and is both sufficient and required for skeletal muscle atrophy. J Cell Sci.

[166] Tao R, Zhang B, Li Y, King JL, Tian R, Xia S, Schiavon CR, Dong JT (2018). HDAC-mediated deacetylation of KLF5 associates with its proteasomal degradation. Biochem Biophys Res Commun.

[167] Matsumura T, Suzuki T, Aizawa K, Munemasa Y, Muto S, Horikoshi M, Nagai R (2005). The deacetylase HDAC1 negatively regulates the cardiovascular transcription factor Kruppel-like factor 5 through direct interaction. J Biol Chem.

[168] Jin J, Iakova P, Jiang Y, Lewis K, Sullivan E, Jawanmardi N, Donehower L, Timchenko L, Timchenko NA (2013). Transcriptional and translational regulation of C/EBPbeta-HDAC1 protein complexes controls different levels of p53, SIRT1, and PGC1alpha proteins at the early and late stages of liver cancer. J Biol Chem.

[169] Ma W, Cai Y, Shen Y, Chen X, Zhang L, Ji Y, Chen Z, Zhu J, Yang X, Sun H (2021). HDAC4 knockdown alleviates denervation-induced muscle atrophy by inhibiting myogenin-dependent atrogene activation. Front Cell Neurosci.

[170] Choi MC, Cohen TJ, Barrientos T, Wang B, Li M, Simmons BJ, Yang JS, Cox GA, Zhao Y, Yao TP (2012). A direct HDAC4-MAP kinase crosstalk activates muscle atrophy program. Mol Cell.

[171] Tian H, Liu S, Ren J, Lee JKW, Wang R, Chen P (2020). Role of histone deacetylases in skeletal muscle physiology and systemic energy homeostasis: implications for metabolic diseases and therapy. Front Physiol.

[172] LaBarge SA, Migdal CW, Buckner EH, Okuno H, Gertsman I, Stocks B, Barshop BA, Nalbandian SR, Philp A, McCurdy CE (2016). p300 is not required for metabolic adaptation to endurance exercise training. FASEB J.

[173] Svensson K, LaBarge SA, Sathe A, Martins VF, Tahvilian S, Cunliffe JM, Sasik R, Mahata SK, Meyer GA, Philp A (2020). p300 and cAMP response element-binding protein-binding protein in skeletal muscle homeostasis, contractile function, and survival. J Cachexia Sarcopenia Muscle.

[174] Chamberlain W, Gonnella P, Alamdari N, Aversa Z, Hasselgren PO (2012). Multiple muscle wasting-related transcription factors are acetylated in dexamethasone-treated muscle cells. Biochem Cell Biol.

[175] Calissi G, Lam EW, Link W (2021). Therapeutic strategies targeting FOXO transcription factors. Nat Rev Drug Discov.

[176] Dansen TB, Smits LM, van Triest MH, de Keizer PL, van Leenen D, Koerkamp MG, Szypowska A, Meppelink A, Brenkman AB, Yodoi J (2009). Redox-sensitive cysteines bridge p300/CBP-mediated acetylation and FoxO4 activity. Nat Chem Biol.

[177] Bertaggia E, Coletto L, Sandri M (2012). Posttranslational modifications control FoxO3 activity during denervation. Am J Physiol Cell Physiol.

[178] Sandri M, Sandri C, Gilbert A, Skurk C, Calabria E, Picard A, Walsh K, Schiaffino S, Lecker SH, Goldberg AL (2004). Foxo transcription factors induce the atrophy-related ubiquitin ligase atrogin-1 and cause skeletal muscle atrophy. Cell.

[179] Senf SM, Dodd SL, Judge AR (2010). FOXO signaling is required for disuse muscle atrophy and is directly regulated by Hsp70. Am J Physiol Cell Physiol.

[180] Fan Z, Wu J, Chen QN, Lyu AK, Chen JL, Sun Y, Lyu Q, Zhao YX, Guo A, Liao ZY (2020). Type 2 diabetes-induced overactivation of P300 contributes to skeletal muscle atrophy by inhibiting autophagic flux. Life Sci.

[181] Sin TK, Zhang G, Zhang Z, Zhu JZ, Zuo Y, Frost JA, Li M, Li YP (2021). Cancer-induced muscle wasting requires p38beta MAPK activation of p300. Cancer Res.

[182] Liu L, Koike H, Ono T, Hayashi S, Kudo F, Kaneda A, Kagechika H, Manabe I, Nakashima T, Oishi Y (2021). Identification of a KLF5-dependent program and drug development for skeletal muscle atrophy. Proc Natl Acad Sci USA.

[183] Moresi V, Carrer M, Grueter CE, Rifki OF, Shelton JM, Richardson JA, Bassel-Duby R, Olson EN (2012). Histone deacetylases 1 and 2 regulate autophagy flux and skeletal muscle homeostasis in mice. Proc Natl Acad Sci USA.

[184] Walsh ME, Van Remmen H (2016). Emerging roles for histone deacetylases in age-related muscle atrophy. Nutr Healthy Aging.

[185] Cohen TJ, Waddell DS, Barrientos T, Lu Z, Feng G, Cox GA, Bodine SC, Yao TP (2007). The histone deacetylase HDAC4 connects neural activity to muscle transcriptional reprogramming. J Biol Chem.

[186] Moresi V, Williams AH, Meadows E, Flynn JM, Potthoff MJ, McAnally J, Shelton JM, Backs J, Klein WH, Richardson JA (2010). Myogenin and class II HDACs control neurogenic muscle atrophy by inducing E3 ubiquitin ligases. Cell.

[187] Gu Y, Lin Y, Li M, Zong C, Sun H, Shen Y, Zhu J (2022). An analysis of lncRNA-miRNA-mRNA networks to investigate the effects of HDAC4 inhibition on skeletal muscle atrophy caused by peripheral nerve injury. Ann Transl Med.

[188] Castets P, Rion N, Theodore M, Falcetta D, Lin S, Reischl M, Wild F, Guerard L, Eickhorst C, Brockhoff M (2019). mTORC1 and PKB/Akt control the muscle response to denervation by regulating autophagy and HDAC4. Nat Commun.

[189] Bruneteau G, Simonet T, Bauche S, Mandjee N, Malfatti E, Girard E, Tanguy ML, Behin A, Khiami F, Sariali E (2013). Muscle histone deacetylase 4 upregulation in amyotrophic lateral sclerosis: potential role in reinnervation ability and disease progression. Brain.

[190] Cohen TJ, Barrientos T, Hartman ZC, Garvey SM, Cox GA, Yao TP (2009). The deacetylase HDAC4 controls myocyte enhancing factor-2-dependent structural gene expression in response to neural activity. FASEB J.

[191] Pigna E, Simonazzi E, Sanna K, Bernadzki KM, Proszynski T, Heil C, Palacios D, Adamo S, Moresi V (2019). Histone deacetylase 4 protects from denervation and skeletal muscle atrophy in a murine model of amyotrophic lateral sclerosis. EBioMedicine.

[192] Dupre-Aucouturier S, Castells J, Freyssenet D, Desplanches D (2015). Trichostatin A, a histone deacetylase inhibitor, modulates unloaded-induced skeletal muscle atrophy. J Appl Physiol (1985).

[193] Walsh ME, Bhattacharya A, Sataranatarajan K, Qaisar R, Sloane L, Rahman MM, Kinter M, Van Remmen H (2015). The histone deacetylase inhibitor butyrate improves metabolism and reduces muscle atrophy during aging. Aging Cell.

[194] Rouaux C, Panteleeva I, Rene F, de Gonzalez Aguilar JL, Echaniz-Laguna A, Dupuis L, Menger Y, Boutillier AL, Loeffler JP (2007). Sodium valproate exerts neuroprotective effects in vivo through CREB-binding protein-dependent mechanisms but does not improve survival in an amyotrophic lateral sclerosis mouse model. J Neurosci.

[195] Prior-Gonzalez M, Lazo-Gomez R, Tapia R (2023). Sodium butyrate does not protect spinal motor neurons from AMPA-induced excitotoxic degeneration in vivo. Dis Model Mech.

[196] Jonkhout N, Tran J, Smith MA, Schonrock N, Mattick JS, Novoa EM (2017). The RNA modification landscape in human disease. RNA.

[197] Fu Y, Dominissini D, Rechavi G, He C (2014). Gene expression regulation mediated through reversible m(6)A RNA methylation. Nat Rev Genet.

[198] Roundtree IA, Evans ME, Pan T, He C (2017). Dynamic RNA modifications in gene expression regulation. Cell.

[199] Zhao X, Yang Y, Sun BF, Shi Y, Yang X, Xiao W, Hao YJ, Ping XL, Chen YS, Wang WJ (2014). FTO-dependent demethylation of N6-methyladenosine regulates mRNA splicing and is required for adipogenesis. Cell Res.

[200] Xiao W, Adhikari S, Dahal U, Chen YS, Hao YJ, Sun BF, Sun HY, Li A, Ping XL, Lai WY (2016). Nuclear m(6)A reader YTHDC1 regulates mRNA splicing. Mol Cell.

[201] Jiang X, Liu B, Nie Z, Duan L, Xiong Q, Jin Z, Yang C, Chen Y (2021). The role of m6A modification in the biological functions and diseases. Signal Transduct Target Ther.

[202] Meyer KD, Jaffrey SR (2017). Rethinking m(6)A readers, writers, and erasers. Annu Rev Cell Dev Biol.

[203] He PC, He C (2021). m(6) A RNA methylation: from mechanisms to therapeutic potential. EMBO J.

[204] Yu B, Liu J, Zhang J, Mu T, Feng X, Ma R, Gu Y (2022). Regulatory role of RNA N(6)-methyladenosine modifications during skeletal muscle development. Front Cell Dev Biol.

[205] Li J, Pei Y, Zhou R, Tang Z, Yang Y (2021). Regulation of RNA N(6)-methyladenosine modification and its emerging roles in skeletal muscle development. Int J Biol Sci.

[206] Han J, Kong H, Wang X, Zhang XA (2022). Novel insights into the interaction between N6-methyladenosine methylation and noncoding RNAs in musculoskeletal disorders. Cell Prolif.

[207] Imbriano C, Moresi V, Belluti S, Renzini A, Cavioli G, Maretti E, Molinari S (2023). Epitranscriptomics as a new layer of regulation of gene expression in skeletal muscle: known functions and future perspectives. Int J Mol Sci.

[208] Petrosino JM, Hinger SA, Golubeva VA, Barajas JM, Dorn LE, Iyer CC, Sun HL, Arnold WD, He C, Accornero F (2022). The m(6)A methyltransferase METTL3 regulates muscle maintenance and growth in mice. Nat Commun.

[209] Xie SJ, Tao S, Diao LT, Li PL, Chen WC, Zhou ZG, Hu YX, Hou YR, Lei H, Xu WY (2021). Characterization of long non-coding RNAs modified by m(6)A RNA methylation in skeletal myogenesis. Front Cell Dev Biol.

[210] Diao LT, Xie SJ, Lei H, Qiu XS, Huang MC, Tao S, Hou YR, Hu YX, Sun YJ, Zhang Q (2021). METTL3 regulates skeletal muscle specific miRNAs at both transcriptional and post-transcriptional levels. Biochem Biophys Res Commun.

[211] Liu Q, Deng J, Qiu Y, Gao J, Li J, Guan L, Lee H, Zhou Q, Xiao J (2021). Non-coding RNA basis of muscle atrophy. Mol Ther Nucleic Acids.

[212] Liang Y, Han H, Xiong Q, Yang C, Wang L, Ma J, Lin S, Jiang YZ (2021). METTL3-mediated m(6)A methylation regulates muscle stem cells and muscle regeneration by notch signaling pathway. Stem Cells Int.

[213] Gheller BJ, Blum JE, Fong EHH, Malysheva OV, Cosgrove BD, Thalacker-Mercer AE (2020). A defined N6-methyladenosine (m(6)A) profile conferred by METTL3 regulates muscle stem cell/myoblast state transitions. Cell Death Discov.

[214] Liu J, Zuo H, Wang Z, Wang W, Qian X, Xie Y, Peng D, Xie Y, Hong L, You W (2023). The m6A reader YTHDC1 regulates muscle stem cell proliferation via PI4K-Akt-mTOR signalling. Cell Prolif.

[215] Qiao Y, Sun Q, Chen X, He L, Wang D, Su R, Xue Y, Sun H, Wang H (2023). Nuclear m6A reader YTHDC1 promotes muscle stem cell activation/proliferation by regulating mRNA splicing and nuclear export. Elife.

[216] Sun J, Zhou H, Chen Z, Zhang H, Cao Y, Yao X, Chen X, Liu B, Gao Z, Shen Y (2023). Altered m6A RNA methylation governs denervation-induced muscle atrophy by regulating ubiquitin proteasome pathway. J Transl Med.

[217] Zou S, Toh JD, Wong KH, Gao YG, Hong W, Woon EC (2016). N(6)-Methyladenosine: a conformational marker that regulates the substrate specificity of human demethylases FTO and ALKBH5. Sci Rep.

[218] Kaur S, Tam NY, McDonough MA, Schofield CJ, Aik WS (2022). Mechanisms of substrate recognition and N6-methyladenosine demethylation revealed by crystal structures of ALKBH5-RNA complexes. Nucleic Acids Res.

[219] Toh JDW, Crossley SWM, Bruemmer KJ, Ge EJ, He D, Iovan DA, Chang CJ (2020). Distinct RNA N-demethylation pathways catalyzed by nonheme iron ALKBH5 and FTO enzymes enable regulation of formaldehyde release rates. Proc Natl Acad Sci USA.

[220] Church C, Moir L, McMurray F, Girard C, Banks GT, Teboul L, Wells S, Bruning JC, Nolan PM, Ashcroft FM (2010). Overexpression of Fto leads to increased food intake and results in obesity. Nat Genet.

[221] De Micheli AJ, Laurilliard EJ, Heinke CL, Ravichandran H, Fraczek P, Soueid-Baumgarten S, De Vlaminck I, Elemento O, Cosgrove BD (2020). Single-cell analysis of the muscle stem cell hierarchy identifies heterotypic communication signals involved in skeletal muscle regeneration. Cell Rep.

[222] Krasniewski LK, Chakraborty P, Cui CY, Mazan-Mamczarz K, Dunn C, Piao Y, Fan J, Shi C, Wallace T, Nguyen C (2022). Single-cell analysis of skeletal muscle macrophages reveals age-associated functional subpopulations. Elife.

[223] Liu Y, Yang Y, Wu R, Gao CC, Liao X, Han X, Zeng B, Huang C, Luo Y, Liu Y (2022). mRNA m(5)C inhibits adipogenesis and promotes myogenesis by respectively facilitating YBX2 and SMO mRNA export in ALYREF-m(5)C manner. Cell Mol Life Sci.

[224] Marasco LE, Kornblihtt AR (2023). The physiology of alternative splicing. Nat Rev Mol Cell Biol.

[225] Cartegni L, Chew SL, Krainer AR (2002). Listening to silence and understanding nonsense: exonic mutations that affect splicing. Nat Rev Genet.

[226] Bartys N, Kierzek R, Lisowiec-Wachnicka J (2019). The regulation properties of RNA secondary structure in alternative splicing. Biochim Biophys Acta Gene Regul Mech.

[227] Singh RN, Singh NN (2018). Mechanism of splicing regulation of spinal muscular atrophy genes. Adv Neurobiol.

[228] Rigillo G, Basile V, Belluti S, Ronzio M, Sauta E, Ciarrocchi A, Latella L, Saclier M, Molinari S, Vallarola A (2021). The transcription factor NF-Y participates to stem cell fate decision and regeneration in adult skeletal muscle. Nat Commun.

[229] Sebastian S, Faralli H, Yao Z, Rakopoulos P, Palii C, Cao Y, Singh K, Liu QC, Chu A, Aziz A (2013). Tissue-specific splicing of a ubiquitously expressed transcription factor is essential for muscle differentiation. Genes Dev.

[230] Kutz LC, Mukherji ST, Wang X, Bryant A, Larre I, Heiny JA, Lingrel JB, Pierre SV, Xie Z (2018). Isoform-specific role of Na/K-ATPase alpha1 in skeletal muscle. Am J Physiol Endocrinol Metab.

[231] Ruas JL, White JP, Rao RR, Kleiner S, Brannan KT, Harrison BC, Greene NP, Wu J, Estall JL, Irving BA (2012). A PGC-1alpha isoform induced by resistance training regulates skeletal muscle hypertrophy. Cell.

[232] Zhang M, Han Y, Liu J, Liu L, Zheng L, Chen Y, Xia R, Yao D, Cai X, Xu X (2020). Rbm24 modulates adult skeletal muscle regeneration via regulation of alternative splicing. Theranostics.

[233] Van Pelt DW, Confides AL, Judge AR, Vanderklish PW, Dupont-Versteegden EE (2018). Cold shock protein RBM3 attenuates atrophy and induces hypertrophy in skeletal muscle. J Muscle Res Cell Motil.

[234] Crawford Parks TE, Ravel-Chapuis A, Bondy-Chorney E, Renaud JM, Cote J, Jasmin BJ (2017). Muscle-specific expression of the RNA-binding protein Staufen1 induces progressive skeletal muscle atrophy via regulation of phosphatase tensin homolog. Hum Mol Genet.

[235] Cox DC, Guan X, Xia Z, Cooper TA (2020). Increased nuclear but not cytoplasmic activities of CELF1 protein leads to muscle wasting. Hum Mol Genet.

[236] Janice Sanchez B, Tremblay AK, Leduc-Gaudet JP, Hall DT, Kovacs E, Ma JF, Mubaid S, Hallauer PL, Phillips BL, Vest KE (2019). Depletion of HuR in murine skeletal muscle enhances exercise endurance and prevents cancer-induced muscle atrophy. Nat Commun.

[237] Alexander MS, Hightower RM, Reid AL, Bennett AH, Iyer L, Slonim DK, Saha M, Kawahara G, Kunkel LM, Kopin AS (2021). hnRNP L is essential for myogenic differentiation and modulates myotonic dystrophy pathologies. Muscle Nerve.

[238] Singh RK, Kolonin AM, Fiorotto ML, Cooper TA (2018). Rbfox-splicing factors maintain skeletal muscle mass by regulating Calpain3 and proteostasis. Cell Rep.

[239] Li M, Zhuang Y, Batra R, Thomas JD, Li M, Nutter CA, Scotti MM, Carter HA, Wang ZJ, Huang XS (2020). HNRNPA1-induced spliceopathy in a transgenic mouse model of myotonic dystrophy. Proc Natl Acad Sci USA.

[240] Shi DL, Grifone R (2021). RNA-binding proteins in the post-transcriptional control of skeletal muscle development, regeneration and disease. Front Cell Dev Biol.

[241] Qiu J, Wu L, Chang Y, Sun H, Sun J (2021). Alternative splicing transitions associate with emerging atrophy phenotype during denervation-induced skeletal muscle atrophy. J Cell Physiol.

[242] Henrich M, Ha P, Wang Y, Ting K, Stodieck L, Soo C, Adams JS, Chun R (2022). Alternative splicing diversifies the skeletal muscle transcriptome during prolonged spaceflight. Skelet Muscle.

[243] Kontrogianni-Konstantopoulos A, Ackermann MA, Bowman AL, Yap SV, Bloch RJ (2009). Muscle giants: molecular scaffolds in sarcomerogenesis. Physiol Rev.

[244] Wang N, Hu Y, Wang Z (2023). Regulation of alternative splicing: Functional interplay with epigenetic modifications and its implication to cancer. Wiley Interdiscip Rev RNA..

[245] Yearim A, Gelfman S, Shayevitch R, Melcer S, Glaich O, Mallm JP, Nissim-Rafinia M, Cohen AH, Rippe K, Meshorer E (2015). HP1 is involved in regulating the global impact of DNA methylation on alternative splicing. Cell Rep.

[246] Luco RF, Pan Q, Tominaga K, Blencowe BJ, Pereira-Smith OM, Misteli T (2010). Regulation of alternative splicing by histone modifications. Science.

[247] Luco RF, Allo M, Schor IE, Kornblihtt AR, Misteli T (2011). Epigenetics in alternative pre-mRNA splicing. Cell.

[248] Rahhal R, Seto E (2019). Emerging roles of histone modifications and HDACs in RNA splicing. Nucleic Acids Res.

[249] Wiedner HJ, Torres EV, Blue RE, Tsai YH, Parker J, Giudice J (2022). SET domain containing 2 (SETD2) influences metabolism and alternative splicing during myogenesis. FEBS J.

[250] Kolathur KK (2021). Role of promoters in regulating alternative splicing. Gene.

[251] Shukla S, Kavak E, Gregory M, Imashimizu M, Shutinoski B, Kashlev M, Oberdoerffer P, Sandberg R, Oberdoerffer S (2011). CTCF-promoted RNA polymerase II pausing links DNA methylation to splicing. Nature.

[252] Marina RJ, Sturgill D, Bailly MA, Thenoz M, Varma G, Prigge MF, Nanan KK, Shukla S, Haque N, Oberdoerffer S (2016). TET-catalyzed oxidation of intragenic 5-methylcytosine regulates CTCF-dependent alternative splicing. EMBO J.

[253] Schor IE, Fiszbein A, Petrillo E, Kornblihtt AR (2013). Intragenic epigenetic changes modulate NCAM alternative splicing in neuronal differentiation. EMBO J.

[254] Hinkle ER, Wiedner HJ, Black AJ, Giudice J (2019). RNA processing in skeletal muscle biology and disease. Transcription.

[255] Wei G, Almeida M, Pintacuda G, Coker H, Bowness JS, Ule J, Brockdorff N (2021). Acute depletion of METTL3 implicates N (6)-methyladenosine in alternative intron/exon inclusion in the nascent transcriptome. Genome Res.

[256] Louloupi A, Ntini E, Conrad T, Orom UAV (2018). Transient N-6-methyladenosine transcriptome sequencing reveals a regulatory role of m6A in splicing efficiency. Cell Rep.

[257] Jara-Espejo M, Fleming AM, Burrows CJ (2020). Potential G-quadruplex forming sequences and N(6)-methyladenosine colocalize at human pre-mRNA intron splice sites. ACS Chem Biol.

[258] Yang Y, Hsu PJ, Chen YS, Yang YG (2018). Dynamic transcriptomic m(6)A decoration: writers, erasers, readers and functions in RNA metabolism. Cell Res.

[259] Adhikari S, Xiao W, Zhao YL, Yang YG (2016). m(6)A: signaling for mRNA splicing. RNA Biol.

[260] Bartosovic M, Molares HC, Gregorova P, Hrossova D, Kudla G, Vanacova S (2017). N6-methyladenosine demethylase FTO targets pre-mRNAs and regulates alternative splicing and 3'-end processing. Nucleic Acids Res.

[261] Zhu ZM, Huo FC, Zhang J, Shan HJ, Pei DS (2023). Crosstalk between m6A modification and alternative splicing during cancer progression. Clin Transl Med.

[262] Kasowitz SD, Ma J, Anderson SJ, Leu NA, Xu Y, Gregory BD, Schultz RM, Wang PJ (2018). Nuclear m6A reader YTHDC1 regulates alternative polyadenylation and splicing during mouse oocyte development. PLoS Genet.

[263] Li S, Qi Y, Yu J, Hao Y, He B, Zhang M, Dai Z, Jiang T, Li S, Huang F (2022). Nuclear Aurora kinase A switches m(6)A reader YTHDC1 to enhance an oncogenic RNA splicing of tumor suppressor RBM4. Signal Transduct Target Ther.

[264] Liu Y, Luo Y, Shen L, Guo R, Zhan Z, Yuan N, Sha R, Qian W, Wang Z, Xie Z (2020). Splicing factor SRSF1 is essential for satellite cell proliferation and postnatal maturation of neuromuscular junctions in mice. Stem Cell Reports.

[265] Li Z, Gilbert JA, Zhang Y, Zhang M, Qiu Q, Ramanujan K, Shavlakadze T, Eash JK, Scaramozza A, Goddeeris MM (2012). An HMGA2-IGF2BP2 axis regulates myoblast proliferation and myogenesis. Dev Cell.

[266] Okamura T, Okada H, Hashimoto Y, Majima S, Senmaru T, Nakanishi N, Asano M, Yamazaki M, Hamaguchi M, Fukui M (2021). Let-7e-5p Regulates IGF2BP2, and Induces Muscle Atrophy. Front Endocrinol (Lausanne).

[267] Gujar H, Weisenberger DJ, Liang G (2019). The roles of human DNA methyltransferases and their isoforms in shaping the epigenome. Genes (Basel).

[268] Aguirre-Arteta AM, Grunewald I, Cardoso MC, Leonhardt H (2000). Expression of an alternative Dnmt1 isoform during muscle differentiation. Cell Growth Differ.

[269] Ostler KR, Davis EM, Payne SL, Gosalia BB, Exposito-Cespedes J, Le Beau MM, Godley LA (2007). Cancer cells express aberrant DNMT3B transcripts encoding truncated proteins. Oncogene.

[270] Gordon CA, Hartono SR, Chedin F (2013). Inactive DNMT3B splice variants modulate de novo DNA methylation. PLoS ONE.

[271] Duymich CE, Charlet J, Yang X, Jones PA, Liang G (2016). DNMT3B isoforms without catalytic activity stimulate gene body methylation as accessory proteins in somatic cells. Nat Commun.

[272] Van Emburgh BO, Robertson KD (2011). Modulation of Dnmt3b function in vitro by interactions with Dnmt3L, Dnmt3a and Dnmt3b splice variants. Nucleic Acids Res.

[273] Zeng Y, Ren R, Kaur G, Hardikar S, Ying Z, Babcock L, Gupta E, Zhang X, Chen T, Cheng X (2020). The inactive Dnmt3b3 isoform preferentially enhances Dnmt3b-mediated DNA methylation. Genes Dev.

[274] Arroyo M, Hastert FD, Zhadan A, Schelter F, Zimbelmann S, Rausch C, Ludwig AK, Carell T, Cardoso MC (2022). Isoform-specific and ubiquitination dependent recruitment of Tet1 to replicating heterochromatin modulates methylcytosine oxidation. Nat Commun.

[275] Verrier L, Escaffit F, Chailleux C, Trouche D, Vandromme M (2011). A new isoform of the histone demethylase JMJD2A/KDM4A is required for skeletal muscle differentiation. PLoS Genet.

[276] Agosto LM, Mallory MJ, Ferretti MB, Blake D, Krick KS, Gazzara MR, Garcia BA, Lynch KW (2023). Alternative splicing of HDAC7 regulates its interaction with 14-3-3 proteins to alter histone marks and target gene expression. Cell Rep.

[277] Zhu LY, Zhu YR, Dai DJ, Wang X, Jin HC (2018). Epigenetic regulation of alternative splicing. Am J Cancer Res.

[278] Xu RY, Ding Z, Zhao Q, Ke TY, Chen S, Wang XY, Wang YY, Sheng MF, Wang W, Long N (2022). An alternatively spliced variant of METTL3 mediates tumor suppression in hepatocellular carcinoma. Genes (Basel).

[279] Poh HX, Mirza AH, Pickering BF, Jaffrey SR (2022). Alternative splicing of METTL3 explains apparently METTL3-independent m6A modifications in mRNA. PLoS Biol.

[280] Lee S, Jung H, Choi S, Cho N, Kim EM, Kim KK (2023). Intron retention decreases METTL3 expression by inhibiting mRNA export to the cytoplasm. BMB Rep.

[281] Chen S, Yang C, Wang ZW, Hu JF, Pan JJ, Liao CY, Zhang JQ, Chen JZ, Huang Y, Huang L (2021). CLK1/SRSF5 pathway induces aberrant exon skipping of METTL14 and Cyclin L2 and promotes growth and metastasis of pancreatic cancer. J Hematol Oncol.

[282] Lois S, Blanco N, Martinez-Balbas M, de la Cruz X (2007). The functional modulation of epigenetic regulators by alternative splicing. BMC Genomics.

[283] Yang X, Mei C, Raza SHA, Ma X, Wang J, Du J, Zan L (2022). Interactive regulation of DNA demethylase gene TET1 and m(6)A methyltransferase gene METTL3 in myoblast differentiation. Int J Biol Macromol.

[284] Zhang D, Wu S, Zhang X, Ren S, Tang Z, Gao F (2022). Coordinated transcriptional and post-transcriptional epigenetic regulation during skeletal muscle development and growth in pigs. J Anim Sci Biotechnol.

[285] Pandorf CE, Haddad F, Wright C, Bodell PW, Baldwin KM (2009). Differential epigenetic modifications of histones at the myosin heavy chain genes in fast and slow skeletal muscle fibers and in response to muscle unloading. Am J Physiol Cell Physiol.

[286] Lin H, Peng H, Sun Y, Si M, Wu J, Wang Y, Thomas SS, Sun Z, Hu Z (2023). Reprogramming of cis-regulatory networks during skeletal muscle atrophy in male mice. Nat Commun.

[287] Wu YL, Lin ZJ, Li CC, Lin X, Shan SK, Guo B, Zheng MH, Li F, Yuan LQ, Li ZH (2023). Epigenetic regulation in metabolic diseases: mechanisms and advances in clinical study. Signal Transduct Target Ther.

[288] Gjaltema RAF, Rots MG (2020). Advances of epigenetic editing. Curr Opin Chem Biol.

[289] Bouyahya A, El Omari N, Bakha M, Aanniz T, El Menyiy N, El Hachlafi N, El Baaboua A, El-Shazly M, Alshahrani MM, Al Awadh AA (2022). Pharmacological Properties of Trichostatin A, focusing on the anticancer potential: a comprehensive review. Pharmaceuticals (Basel).

